# NF-κB/c-Rel deficiency causes Parkinson’s disease-like prodromal symptoms and progressive pathology in mice

**DOI:** 10.1186/s40035-019-0154-z

**Published:** 2019-05-21

**Authors:** Edoardo Parrella, Arianna Bellucci, Vanessa Porrini, Marina Benarese, Annamaria Lanzillotta, Gaia Faustini, Francesca Longhena, Giulia Abate, Daniela Uberti, Marina Pizzi

**Affiliations:** Department of Molecular and Translational Medicine, University of Brescia, Viale Europa 11, 25123 Brescia, Italy

**Keywords:** Parkinson’s disease, NF-κB/c-Rel, non-motor symptoms, α-synuclein, pathology progression

## Abstract

**Background:**

Parkinson’s disease (PD), the most common neurodegenerative movement disorder, is characterized by dopaminergic nigrostriatal neuron loss and brain accumulation of Lewy bodies, protein aggregates mainly composed of α-synuclein. We reported that mice deficient for NF-κB/c-Rel (c-rel^-/-^) develop a late-onset parkinsonism. At 18 months of age, c-rel^-/-^ mice showed nigrostriatal degeneration and accumulation of α-synuclein aggregates associated with a motor impairment responsive to L-DOPA administration. Being c-Rel protein a transcriptional regulator for mitochondrial anti-oxidant and antiapoptotic factors, it has been inferred that its deficiency may affect the resilience of “energy demanding” nigral dopaminergic neurons to the aging process.

PD patients manifest a prodromal syndrome that includes olfactory and gastrointestinal dysfunctions years before the frank degeneration of nigrostriatal neurons and appearance of motor symptoms. According to the Braak staging, the onset of non-motor and motor symptoms relates to progressive ascendant diffusion of α-synuclein pathology in the brain. The aim of this study was to identify whether c-rel^-/-^ deficiency is associated with the onset of premotor signs of PD and spatio-temporal progression of cerebral α-synuclein deposition.

**Methods:**

Intestinal and olfactory functions, intestine and brain α-synuclein deposition as well as striatal alterations, were assessed in c-rel^-/-^ and control mice from 2 to 18 months of age.

**Results:**

From 2 months of age, c-rel^-/-^ mice displayed intestinal constipation and increasing olfactory impairment. At 2 months, c-rel^-/-^ mice exhibited a mild α-synuclein accumulation in the distal colon. Moreover, they developed an age-dependent deposition of fibrillary α-synuclein that, starting at 5 months from the olfactory bulbs, dorsal motor nucleus of vagus and locus coeruleus, reached the substantia nigra at 12 months. At this age, the α-synuclein pathology associated with a drop of dopamine transporter in the striatum that anticipated by 6 months the axonal degeneration. From 12 months onwards oxidative/nitrosative stress developed in the striatum in parallel with altered expression of mitochondrial homeostasis regulators in the substantia nigra.

**Conclusions:**

In c-rel^-/-^ mice, reproducing a parkinsonian progressive pathology with non-motor and motor symptoms, a Braak-like pattern of brain ascending α-synuclein deposition occurs. The peculiar phenotype of c-rel^-/-^ mice envisages a potential contribution of c-Rel dysregulation to the pathogenesis of PD.

**Electronic supplementary material:**

The online version of this article (10.1186/s40035-019-0154-z) contains supplementary material, which is available to authorized users.

## Background

Parkinson’s disease (PD), the most common movement disorder, is characterized by abnormal α-synuclein deposition in fibrillary aggregates composing intraneuronal inclusions that are called Lewy bodies (LB). In PD patients, LB pathology involves numerous brain areas (i.e. the substantia nigra (SN), olfactory bulbs (OB), dorsal motor nucleus of the vagus (DMV), locus coeruleus (LC), nucleus basalis of Meynert, hypothalamus, cerebral cortex, cranial nerve motor nuclei). Peripheral nerves of the autonomic nervous system innervating the heart, gut, submandibular glands, pharyngeal muscles, skin and vagal preganglionic projections can also be affected [1, 2].

Besides classical motor symptoms, PD patients can manifest a plethora of typical non-motor symptoms such as constipation, impaired olfaction, anxiety, depression, excessive daytime sleepiness and rapid eye movement sleep behaviour disorder (RBD) [3]. Most, if not all, of the non-motor features usually occur years before the onset of motor symptoms [4] and are considered prodromal signs of the disease [5].

It is widely assumed that brain α-synuclein deposition is central to PD pathogenesis. This was supported by Braak and collaborators that, from the analysis of post-mortem PD patients’ brains at different disease stages, proposed a correlation between the progression of symptoms and the topographical pattern of LB diffusion [6–9]. Six neuropathological stages of PD have been identified. In stage 1, α-synuclein pathology is confined to the DMV as well as olfactory structures and affected subjects exhibit non-motor symptoms such as hyposmia and constipation [10]. Depression, anxiety and RBD have been suggested to appear between stage 1 and stage 2 when α-synuclein immunoreactivity becomes detectable in LC [10]. At stage 3, α-synuclein accumulates in the SN, amygdala and nucleus of Meynert, while it progresses to mesocortical areas at stage 4 [6–8]. The motor symptoms, allowing the disease diagnosis, manifest between stages 3 and 4 [10]. Finally, at stages 5 and 6, when the cognitive disturbances may occur [10], α-synuclein pathology affects temporal mesocortex and neocortical areas, respectively.

To-date, the lack of reliable tracer for longitudinal evaluation of α-synuclein deposition in the brain has hampered the achievement of clear-cut evidence demonstrating that the anatomical progression of α-synuclein pathology dictates typology and onset of PD symptoms [11]. Hence, the staging proposed by Braak still deserves a validation. Animal models able to reproduce both premotor symptoms and progressive pathology of PD would be extremely useful to this aim [12].

We recently showed that mice deficient for NF-κB/c-Rel protein (c-rel^-/-^ mice) model an aging-related mild PD phenotype [13]. At 18 months of age, c-rel^-/-^ mice exhibit a 40% loss of dopaminergic neurons and accumulation of α-synuclein aggregates in the SN pars compacta. The mice also display a 60% reduction of striatal dopaminergic fibers and decreased dopamine content, as well as increased levels of divalent metal transporter 1 (DMT1) and iron in SN pars compacta and striatum. These alterations are accompanied by L-DOPA-reversible hypomotility and gait-related deficits [13, 14]. The nigral neurodegeneration in c-rel^-/-^ mice are anticipated, at 12 months, by a mild and transient neuroinflammation state, as revealed by the transcription analysis of cytokines and microglia /macrophage activation genes [15] and the lack of astrogliosis [13, 15]. A finding in line with evidence demonstrating normal CSF cytokines level in patients affected by PD, when it is not associated with severe symptoms of depression, anxiety, fatigue, and cognition [16, 17].

In this study, we investigated whether the c-Rel deficiency is also able to trigger symptoms and pathology peculiar of prodromal PD. In particular, we studied whether c-rel^-/-^ mice develop constipation and olfactory dysfunctions, as well as caudal-rostral progression of α-synuclein deposition in the brain with alterations of striatal dopaminergic terminals, preceding the onset of motor symptoms.

We found that starting from early premotor stages (2 months of age), c-rel^-/-^ mice exhibit intestinal deficits and hyposmia. In 2-month-old c-rel^-/-^ mice, α-synuclein is mildly accumulated in the myenteric ganglia of distal colon. From 5 months, the non-motor symptoms were accompanied by accumulation of aggregated α-synuclein in the DMV, LC and OB. From 12 months, the aggregation of α-synuclein affected the SN pars compacta. A loss of dopamine transporter (DAT) and increase of oxidative/nitrosative stress in the striatum also became evident at 12 months, an age when mice show neither loss of nigral dopaminergic cells nor motor deficits yet [13]. The striatal degeneration was paralleled by the altered expression of proteins governing mitochondrial homeostasis in the SN.

Our data show that, in a mouse model able to recapitulate progressive PD-like symptoms and neuropathology, the evolution of α-synuclein deposition follows the anatomical staging proposed by Braak. This evidence suggests a potential pathogenic role of c-Rel dysregulation in sporadic PD onset and progression that warrants further investigation.

## Methods

### Experimental animals

C57BL/6 mice carrying the c-Rel gene null mutation (c-rel^-/-^) were originally generated by inserting the neomycin cassette into the fifth exon of the c-Rel gene [18]. Both c-rel^-/-^ and c-rel^+/+^ wild-type (wt) mice were housed in the animal facility of the Department of Molecular and Translational Medicine of the University of Brescia [13]. Animals were maintained in individual ventilated cages under 12h/12h light/dark cycles with access to standard rodent food and water *ad libitum*. The cages were enriched with nesting material and mouse houses red (Tecniplast). Mice were housed in groups of 2-4/cage unless specified differently. Humidity and room temperature were maintained at 55% and 22–23°C, respectively. All animal studies were approved by the Animal-welfare body of the University of Brescia and were in accordance with the Directive 2010/63/EU on the protection of animals used for scientific purposes. All the procedures performed accomplished the ethical standards of the University of Brescia. Only male mice were used in this study.

### Behavioral studies

#### Colon motility

Colon motility was assessed by one-hour stool collection assay [19]. The tests were performed in a dedicated quiet room during the light phase at the same time every day (between 10:00 and 12:00 AM). Each mouse was removed from its home cage and placed in a clean, empty plastic cage [36 cm (length) x 15.5 cm (width) x 13.5 cm (height)] without food and water for one hour. Stool pellets were collected immediately after expulsion and placed in a pre-weighed sealed 1.5 mL microtube (Biosigma). The number of pellets expulsed by each mouse was recorded. Stool frequency was expressed as pellets/hour normalized per 30 grams mouse body weight. The tubes were weighed to obtain the wet weight of the stool. The pellets were then dried overnight at 65 °C and reweighed to obtain the dry weight. Stool water content percentage was calculated as difference between wet and dry stool weight over the wet stool weight.

#### Food and water intake

Food and water intake were determined the days following the one-hour stool collection assay [20]. Mice were individually housed in cages provided with a pre-weighed amount of rodent chow and a pre-measured volume of water in the drinking bottle. Mouse body weight and the remaining quantities of chow and water were measured at the same time every day for the following two consecutive days. Food and water intake were calculated as average consumption over two days normalized per 30 grams mouse body weight.

#### Open field

Anxiety status was determined in a black plastic open field box (40 x 40 x 40 cm) virtually divided in a peripheral and central zone of identical area [21]. By using a video tracking system (Ugo Basile), we monitored the time spent by the mice in the central area for 5 minutes. A reduced time in the central zone is associated with higher anxiety levels. The task was performed during the dark phase.

#### Odor detection test

The threshold of odor detection was evaluated according to the protocol described by Petit and colleagues [22]. The task was performed during the light phase in a dedicated quiet room. Briefly, the mice were placed in an empty plastic cage [36 cm (length) x 15.5 cm (width) x 13.5 cm (height)] containing two cartridges, one filled with water and the other filled with vanilla extracts (Erba Vita) diluted to the concentrations 1:10^8^, 1:10^6^ or 1:10^4^. The cartridge consisted of a plastic tube (1.5 ml microtube, Biosigma) cut at the two extremities and filled with a piece of compress that was not accessible to the mice. Every daily set of tests, odor dilutions were prepared fresh and 400 μl of them were applied to the compress (200 μl each side of the cartridge). The test consisted of three sessions of 5 minutes each distributed in three consecutive days in which the mice were exposed to increasing odor concentrations. During the olfactory tests, mice behavior was recorded using a video-tracking system (Ugo Basile). The time spent by the mice sniffing the cartridges was then scored manually by an operator blind to mice identity, considering any physical contact of the nose or whiskers with the object and/or approach with obvious orientation to it within 2 cm. The results were plotted as percentage of time sniffing the odor, a measure of odor preference, and as total sniffing time, a measure of exploratory behavior. The percentage of time sniffing the odor was calculated as the time the animals spent sniffing the cartridge containing the scent of vanilla to the total time spent sniffing both the cartridges. The total sniffing time was calculated as sum of the seconds spent by the mice sniffing the two cartridges.

#### Odor and item discrimination test

The ability of mice to discriminate between odors and items was assessed by the odor and item discrimination test [22] (Fig. 2c). The task was performed during the light phase in a dedicated quiet room. Briefly, the task consisted of six habituation trials where the mice were placed in an empty plastic cage [36 cm (length) x 15.5 cm (width) x 13.5 cm (height)] containing four cartridges filled with vanilla extract (Erba Vita, familiar odor, F). In the seventh trial (odor discrimination), the mice had to detect that one cartridge had been replaced by an identical one containing orange scent (Flora s.r.l., novel odor, N). In the eighth trial (item discrimination), the usual cartridge containing the novel odor was replaced by a novel item (a different type of cartridge filled with the same orange scent). Trials lasted 2 minutes each and were separated by 1-minute intervals. Mice behavior was recorded during the odor and item discrimination trials using a video-tracking system (Ugo Basile). The time spent sniffing and exploring the cartridges by each mouse was then scored manually by an operator blind to rodents’ identity. Mice that were able to recognize the novel odor or the novel item spent more time sniffing or exploring it.

#### Odor preference test

The odor preference test is based on the protocol described by Petit et al. [22]. The task was performed during the light phase in a dedicated quiet room using an empty plastic cage [36 cm (length) x 15.5 cm (width) x 13.5 cm (height)]. The test consisted of a single trial of 5 minutes during which we exposed mice to two cartridges, one filled with vanilla extract (Erba Vita) and one with orange extract (Flora s.r.l.), both diluted to the concentration 1:10^4^. The time spent sniffing each odor was scored by a researcher blind to mice identity examining the tests recorded by a video-tracking system (Ugo Basile).

### Immunohistochemistry

Mice were anaesthetized with chloral hydrate (400 mg/kg intraperitoneally, Sigma-Aldrich) and transcardially perfused with PBS (Sigma-Aldrich) and 4% (w/v) ice-cold paraformaldehyde (Immunofix, Bio-Optica). Brains were collected, post-fixed and conserved in 30% sucrose. Coronal slices (30 μm or 10 μm thick) were cut with a cryostat to obtain serial sections of the following cerebral areas using bregma-based coordinates [23]: DMV (anterior-posterior –7.43 to –7.67 mm), LC (anterior-posterior –5.41 to – 5.51 mm), SN (anterior-posterior 2.54 to 3.40 mm), striatum (anterior-posterior 1.70 to 2.30 mm) and OB (anterior-posterior 4.25 to 3.89 mm).

Nissl staining was performed by incubating the sections (10 μm thickness) in 0.5% cresyl violet (Sigma-Aldrich). Sections were dehydrated and defatted in xylene and mounted with Eukitt (Calibrated Instruments).

Double immunofluorescence staining α-synuclein/tyrosine hydroxylase (TH) or α-synuclein/choline acetyl transferase (ChAT) was performed in sections (30 μm) incubated with anti-α-synuclein antibody (Syn-1; 1:500, BD Biosciences) overnight at 4 °C, followed by secondary antibody conjugated with Cy3 (1:3000, Jackson ImmunoResearch) for 1 hour at room temperature. Slices were then incubated with a primary anti-TH (1:200, Millipore) or anti-ChAT (1:200, Chemicon) antibody overnight at 4 °C followed by 1 h incubation with Alexa Fluor™ 488-conjugated secondary antibodies (1:400, Jackson ImmunoResearch).

Double immunofluorescence staining for Pser129-α-synuclein/TH or Pser129-α-synuclein/ChAT was performed on 30 μm cryostat sections. Briefly, these were incubated with anti- Pser129-α-synuclein antibody (1:300, Abcam) overnight at 4 °C, washed, and then with a secondary antibody conjugated with Cy3 (1:3000, Jackson ImmunoResearch) for 1 hour at room temperature. Slices were then incubated with a primary anti-TH (1:200, Millipore) or anti-ChAT (1:100, Chemicon) antibody overnight at 4 °C, washed, and then exposed to 1 h incubation with Alexa Fluor™ 488-conjugated secondary antibodies (1:500, Jackson ImmunoResearch).

Double immunofluorescence staining TH/vesicular monoamine transporter 2 (VMAT2) was performed in sections (30 μm) incubated with anti- ΤΗ antibody (Millipore, 1:600) overnight at 4 °C followed by the Alexa Fluor™ 488-conjugated secondary antibody (1:1500, Jackson ImmunoResearch) for 1 hour at room temperature. Slices were then incubated at room temperature with the second primary antibody anti-VMAT2 (1:300, SYnaptic SYstem) for 2 hours, followed by incubation with the biotinylated secondary antibody for 1 hour (1:1000; Vector Laboratories) and finally with the fluorochrome-conjugated streptavidin (Streptavidin 594; 1:1000, Thermo fisher).

For thioflavin S/α-synuclein double staining, sections (30 μm) were incubated in a high-concentration PO_4_ buffer (411mM NaCl, 8.1mM KCl, 30mM NaHPO_4_, 5.2 mM KH_2_PO_4_) pH 7.2. After washing, thioflavin S (Sigma-Aldrich) staining and α-synuclein immunolabelling were performed according to previously described protocols [24]. Some sections were pretreated with proteinase K (20 μg/ml, Invitrogen) in proteinase K buffer containing 10 mM Tris–HCl, pH 7.8, 100 mM NaCl, 0.1% NP40 at 37°C for 5 minutes [25].

Different groups of 2-month-old wt and c-rel^-/-^ mice were sacrificed by cervical dislocation. The colon of mice was removed, cleaned with PBS (Sigma-Aldrich), fixed with 4% (w/v) ice-cold paraformaldehyde (Immunofix, Bio-Optica) for 2 hours and then transferred to 30% sucrose. Alpha–synuclein and βIII-tubulin immunolabeling coupled with thioflavin S and TO-PRO-3 staining was performed on cryostat coronal slices (20 μm thick) of the distal colon mounted on Superfrost slides (Thermo Scientific). Sections were first incubated with thioflavin S (Sigma-Aldrich) in a high-concentration PO_4_ buffer [21]. After washing, slices were incubated with anti- α-synuclein (Syn-1; 1:500, BD Biosciences) and anti- βIII-tubulin (1:300, Sigma-Aldrich) antibodies overnight at 4 °C, washed and then exposed to Cy3 (1:3000, Jackson ImmunoResearch) and Alexa Fluor™ 405 (1:2500, Millipore) conjugated secondary antibodies for 1 hour at room temperature. Finally, cell nuclei were stained by incubating the sections with TO-PRO-3 (1:1000, Thermo Fisher) for 1 minute. Coverslips were then mounted by using Vectashield mounting medium (Vector Laboratories).

Double fluorescence labeling of α-synuclein with either TH, ChAT and Thioflavin S and were examined with a Zeiss, LSM 510 META confocal microscope (Carl Zeiss), with the laser set at 543 or 555 nm in the case of TH and ChAT or 450-543 in the case of Thioflavin S staining. Quadruple thioflavin S/α-synuclein/βIII-tubulin/TO-PRO-3 fluorescence labeling was examined by using a Zeiss LSM 510 META confocal microscope (Carl Zeiss). Double immunofluorescence TH/VMAT and Pser129-α-synuclein with either TH or ChAT were acquired by using a Zeiss LSM880 Confocal microscope with the laser set at 488 and 543 nm. During all the confocal images acquisitions the height of section scanning was 1 μm. Images (512x512 or 1024x1024 pixels) were then re-constructed using LSM Zen Blue Image Examiner (Carl Zeiss) and Adobe Photoshop 7.0 software. In the double labeling with α-synuclein both ChAT and TH images were acquired in blue as a false color. In the quadruple fluorescence labeling of intestine sections TO-PRO-3 images were acquired in orange/yellow as false color.

3,3’-Diaminobenzidine (DAB) immunostaining was performed on free-floating sections (30 μm) using primary antibodies: anti- TH (1:400, Millipore); anti- α-synuclein (Syn-1; 1:500, BD Biosciences); anti-DAT (1:200, Santa Cruz Biotechnology). Brain sections were incubated with biotinylated secondary antibodies (1:800, Vector Laboratories) and visualized by avidin-biotin-horseradish peroxidase technique (ABC Elite; Vector Laboratories) using 0.025% DAB (Sigma-Aldrich) as chromogen. The OB sections were also Nissl-counterstained.

The optical density of striatal TH- and DAT-positive fibers was examined from digitized images using Image-ProPlus software (version 6.2, Media Cybernetics). Brains from 3-6 mice (4 sections from each mouse) were analyzed by examining an average of 6 fields per section.

Quantification of α-synuclein immunoreactivity in the brain and in the distal colon was performed on digitized images using the FIJI (NIH) software. Brains from 3-8 mice (4 sections from each mouse) were analyzed by examining an average of 6 fields per section. For the distal colon analysis we measured the total α-synuclein surface that was then normalized *vs* the enteric ganglion area [26]. For this study, 3-6 sections from 5-6 mice, with an average of 6 fields per section were analyzed.

### Real-time quantitative reverse transcription-polymerase chain reaction (qRT-PCR)

Total RNA was purified from SN using the RNeasy Mini Kit for total RNA extractions (Qiagen). RNA (1 μg) was reverse transcribed using the Quantitect® Reverse Transcription Kit (Qiagen) according to manufacturer instructions. Retrotranscribed cDNA was amplified in 25 μl SYBR Green real-time PCR reactions containing 2–8 μl of cDNA, 12.5 μl of 2× iQ™ SYBR Green Supermix (Bio-Rad), and 1 μl of each 10 μM optimized forward and reverse primers in 8.5–2.5 μl RNase-free water. PCR reaction was performed using a 3-stage program: 3 min at 50 °C, 10 min at 95 °C and 40 cycles of 30 s at 94 °C and 45 s at 60 °C. Incorporation of the SYBR Green dye into the PCR products was monitored in real-time with a BIORAD iCycler detection system, allowing the determination of the threshold cycle (CT) at which the exponential amplification of PCR products began. Each reaction was performed in triplicate. For standardization of quantification, β-actin was amplified simultaneously. The oligonucleotide sequences of the primers used are as follow:

- α-synuclein (*Snca*): For GGCCAAGGAGGGAGTTGT; Rev GCTCCCTCCACTGTCTTCTG
- Mitochondrial uncoupling protein 4 (UCP4) *(Slc25a14)*: For TCCTGACTTGCTGCTGAATG; Rev GGAGTCGGGTTTTTGTGAGA
- Mitochondrial uncoupling protein 5 (UCP5) *(Slc25a27)*: For CGCCTCCCTTCTCTCTACG; Rev TAGTCGTGGCTCTGGGAAAG
- Manganese-dependent Superoxide dismutase (MnSOD) *(Sod2):* For *ACACATTAACGCGCAGATCA ;* Rev *CCTCCAGCAACTCTCCTTTG*
- Peroxisome proliferator-activated receptor gamma coactivator 1-alpha (PGC1α) *(Ppargc1a):* For TCTGGGTGGATTGAAGTGGT; Rev AAATGAGGGCAATCCGTCTT
- B-cell lymphoma-extra large (Bcl-xL) *(Bcl2l1):* For *AGGCAGGCGATGAGTTTGAA;* Rev: *TGAAGCGCTCCTGGCCTTTC*
- β-actin (*Actb*): For GGCTCTTTTCCAGCCTTCCT; Rev ATGCCTGGGTACATGGTGGT.

### Immunoblot analysis

We analyzed striatum levels of DAT by western blot technique. Briefly, striatum tissue was collected, transferred to protease/phosphatase inhibitors-supplemented ice-cold buffer C (320 mM sucrose, 1 mM HEPES, 1 mM MgCl_2_, 10 mM NaHCO_3_, pH 7.4) and sonicated. Homogenates were centrifuged at 13,000 g for 15 minutes and supernatant containing the cytosolic fraction collected. Cytosolic extracts (40 μg protein/sample) were resolved by 4%–12% SDS PAGE gel and transferred to a nitrocellulose membrane (Amersham). Membranes were then incubated with either anti-DAT (1:200, Santa Cruz Biotechnology) or anti-β-actin (1:1000, Sigma Aldrich) primary antibody and secondary antibodies coupled to horseradish peroxidase (1:1500, Santa Cruz Biotechnology). Immunopositive bands were visualized by enhanced chemiluminescence detection reagents (GE Healthcare). Gel analysis was performed using the Gel Pro.3 analysis software (MediaCybernetics).

### 3-nitrotyrosine analysis

Measuring 3-nitrotyrosine (3-NT) indirectly provides an estimation of peroxynitrite (ONOO^−^) radicals, resulted from the reaction of nitric oxide (•NO) with superoxide (O_2_^•−^). 3-NT modified protein levels were determined using the commercially available 3-nitrotyrosine competitive ELISA kit (Abcam). Fifty μL of standards and 15 μg of protein extract derived from striatum were processed following kit manufacturer’s instructions. The degree of competition was proportional to the concentration of soluble 3-NT modified protein in the samples. Data were expressed as ng of 3-NT modified proteins over μg of total protein extract.

### Statistical analysis

Statistical analysis was performed with the GraphPad Prism program. Data were expressed as mean ± SEM (standard error of the mean). Statistical significance was accepted at the 95% confidence level (*P*<0.05). One-hour stool collection assay, odor detection test and 3-nitrotyrosine levels were analyzed using a two-way ANOVA followed by the Bonferroni *post hoc* test. One-sample t-test was used in odor detection test to compare the percentage of time sniffing the odor to chance level (50%). Comparisons between two groups were performed using the parametric two-tailed unpaired Student’s t-test or the nonparametric Kolmogorov-Smirnov test.

## Results

### Reduced colon motility in young c-rel^-/-^ mice

**Fig. 1:**
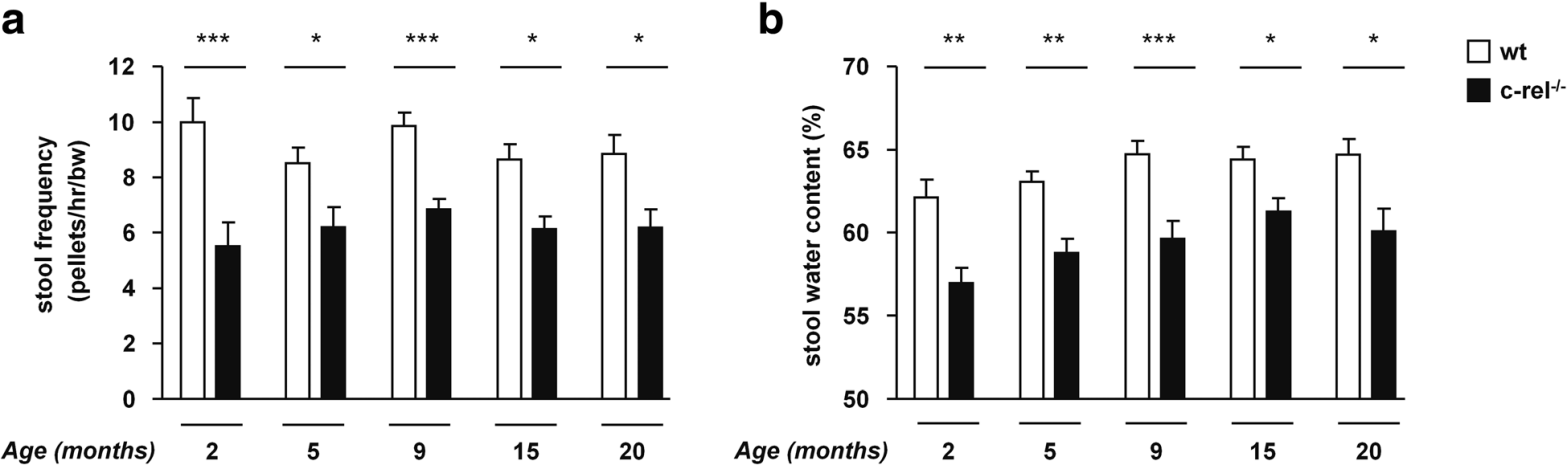
Premotor intestinal dysfunctions in c-rel^-/-^ mice. Stool frequency normalized for 30 grams of body weight (bw) (**a**) and stool water content percentage (**b**) of 2-, 5-, 9-, 15- and 20-month-old wt and c-rel^-/-^ mice are shown (2 months: *n* = 14-15; 5 months: *n* = 19-21; 9 months: *n* = 26-28; 15 months: *n* = 26-28; 20 months: *n* = 13-16). Stool frequency and water content percentage are reduced in c-rel^-/-^ mice at all considered ages. *p<0.05; **p<0.01; ***p<0.001, two way ANOVA followed by Bonferroni *post hoc* test

Colon motility was assessed in both wt and c-rel^-/-^ mice by one-hour stool collection assay using different cohorts of mice at 2, 5, 9, 15 and 20 months of age. We found that stool frequency (normalized against body weight) was significantly lower in c-rel^-/-^ mice, when compared to wt mice, starting from 2 months (Fig. 1a). Stool water content, which is inversely proportional to transit time in the colon segment, was concomitantly diminished in c-rel^-/-^ mice when compared to age-matched wt mice (Fig. 1b). Notably, we observed that the reduction of colon motility was neither dependent on differences in food or water intake nor on alterations in the anxiety status, as these parameters were not decreased in c-rel^-/-^ mice (Additional file 1: Figure S1a-f).

### Early-onset and progressive olfactory impairment in c-rel^-/-^ mice

In order to investigate the presence of olfactory deficits, wt and c-rel^-/-^ mice were subjected to a battery of behavioral tests.

Different cohorts of either wt or c-rel^-/-^ mice were tested for their olfactory threshold by the odor detection test at 2, 5, 9, 12 and 20 months of age. This is based on natural rodents’ behavior to explore odors. The task determines whether animals can detect odors by comparing the time they spend sniffing two cartridges, one filled with water and the other filled with a vanilla extract. Mice with an intact sense of smell instinctively spend more than 50% of time (chance level) sniffing the cartridge containing vanilla extract, while mice affected by olfactory dysfunction do not show preference for any of the two cartridges (percentage of time sniffing the odor similar to the level of chance) [22].

Neither wt nor c-rel^-/-^ mice at all ages were able to detect the lowest odor concentration (dilution 1:10^8^, *p*>0.05, one-sample t-test *vs* chance level, Additional file 1: Figure S1g).

**Fig. 2:**
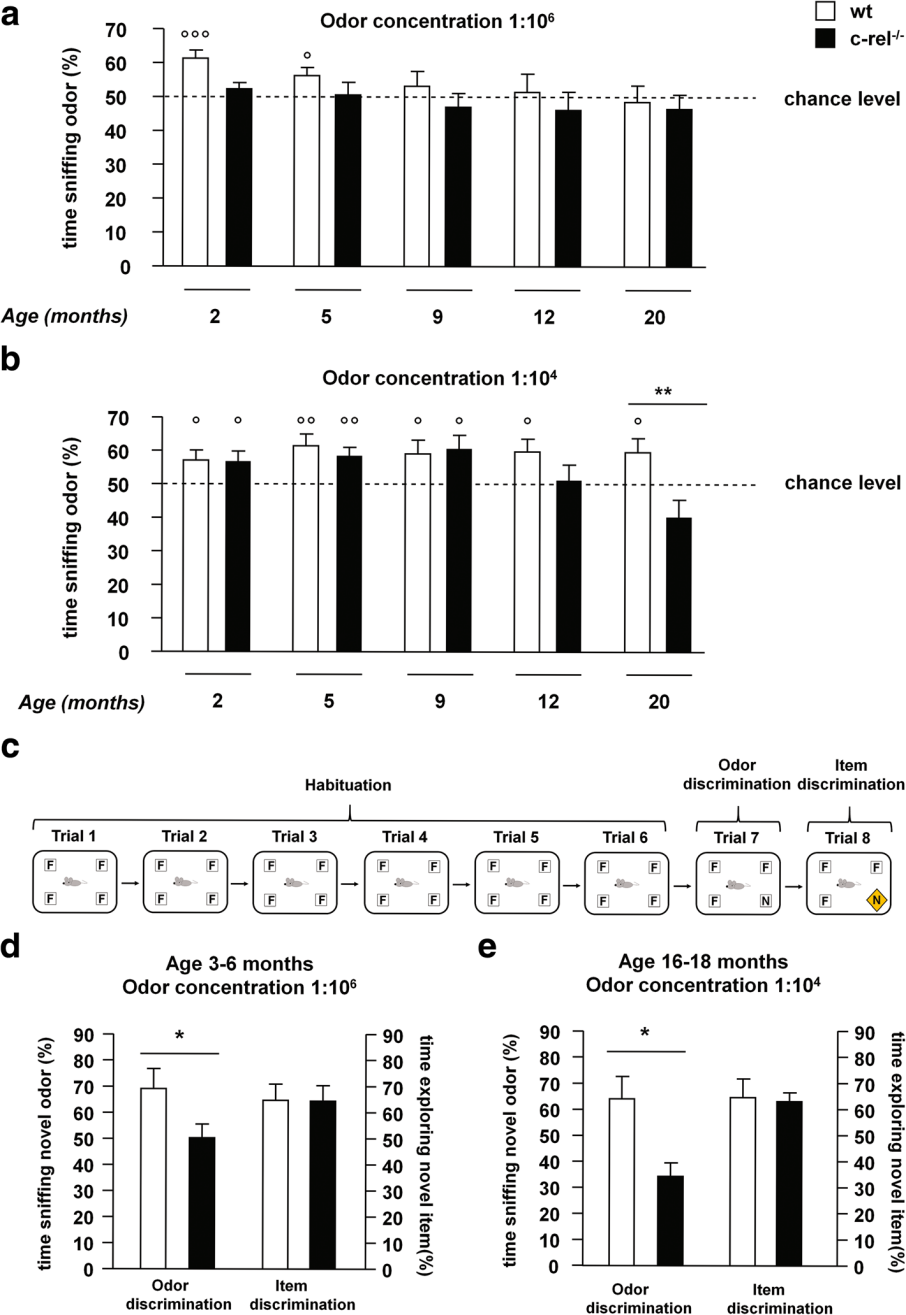
Premotor olfactory dysfunctions in c-rel^-/-^ mice. **a, b** Odor detection test was performed on 2-, 5-, 9-, 12- and 20-month-old wt and c-rel^-/-^ mice (2 months: *n* = 15-18; 5 months: *n* = 18; 9 months: *n* = 10-15; 12 months: *n* = 16-18; 20 months: *n* = 13). The percentage of time sniffing the odor for the different scent dilutions is shown. **a** Odor concentration 1:10^6^. Wild-type mice could locate the odor at 2 and 5 months when the percentage of time sniffing the odor was significantly different from the 50% chance level (°°°*p*<0.001 and °*p*<0.05 respectively, one-sample t-test), whereas c-rel^-/-^ could not (*p*>0.05, one-sample t-test). Neither wt nor c-rel^-/-^ mice could target the odor at 9, 12 and 20 months (*p*>0.05, one-sample t-test). **b** Odor concentration 1:10^4^. Wild-type mice maintained their ability to target the odor through all the considered ages (°*p*<0.05; °°*p*<0.01, one-sample t-test). In contrast, c-rel^-/-^ mice were able to locate the odor till the age of 9 months (°*p*<0.05; °°*p*<0.01, one-sample t-test) and were impaired at 12 and 20 months (*p*>0.05, one-sample t-test). Moreover, 20-month-old c-rel^-/-^ mice displayed a significant odor detection deficit compared to age-matched wt (**p<0.01, two way ANOVA followed by Bonferroni *post hoc* test). **c** Odor and item discrimination test: the task consisted of six habituation trials (habituation) where mice were exposed to four cartridges containing a familiar odor (F, vanilla extract). In the seventh trial (odor discrimination), one cartridge is replaced with an identical one filled with a novel odor (N, orange extract). In the eighth trial (item discrimination), the usual cartridge containing the novel odor was replaced by a novel item (a different type of cartridge filled with the same orange scent). Trials were separated by 1 minute, each trial lasted 2 minutes. **d, e** Odor and item discrimination test was performed on wt and c-rel^-/-^ mice of 3-6 months and 16-18 months of age using odors diluted at concentrations 1:10^6^ and 1:10^4^, respectively (3-6 months: *n* = 9-11; 16-18 months: *n* = 7-8). Percentage of time sniffing the novel odor during the odor discrimination trial and percentage of time exploring the novel item in the item discrimination trial are shown. Mice lacking c-Rel displayed an impaired odor discrimination compared with wt mice at both ages (*p<0.05, t-test). By contrast, both the mice groups spent a similar time exploring the novel item, indicating a proper cognitive performance of the animals in this test (*p*>0.05, t-test)

At the medium vanilla concentration (dilution 1:10^6^, Fig. 2a), 2- and 5-month-old wt mice could locate the odor (*p*<0.001 and *p*<0.05 respectively, one-sample t-test *vs* chance level), whereas age-matched c-rel^-/-^ mice did not (*p*>0.05, one-sample t-test *vs* chance level). At 9, 12 and 20 months, neither wt nor c-rel^-/-^ mice were able to recognize the scent (*p*>0.05, one-sample t-test *vs* chance level).

Up to 9 months of age, both wt and c-rel^-/-^ mice were able to detect the highest vanilla concentration (dilution 1:10^4^, Fig. 2b; *p*< 0.05 and 0.01, one-sample t-test *vs* chance level). Wild type mice maintained their ability to target this odor concentration up to 20 months, whereas c-rel^-/-^ mice resulted impaired from 12 months onwards (*p*>0.05 *vs* chance level, one-sample t-test). The percentage of time spent by 20-month-old c-rel^-/-^ mice to sniff the odor was significantly lower than that of age-matched wt mice (Fig. 2b, *p*<0.01, two way ANOVA followed by Bonferroni *post hoc* test). Although, the total sniffing time did not differ between wt and c-rel^-/-^ mice, indicating a similar exploratory behavior between the two groups during this task (Additional file 1: Figure S1h, 1i).

On the bases of these results, two different cohorts of mice were tested with the odor and item discrimination test (Fig. 2d and e). Young wt and c-rel^-/-^ mice (3-6 months) were challenged to discriminate between familiar and novel odor (vanilla and orange extracts, respectively) at the concentration 1:10^6^ (Fig. 2d), whereas aged animals (16-18 months) were tested with the odors diluted 1:10^4^ (Fig. 2e). We found that the c-rel^-/-^ mice were impaired in recognizing the novel odor at both ages. Conversely, both young and aged c-rel^-/-^ mice were able to recognize the novel item likewise wt controls (Fig. 2d and e), indicating a proper cognitive performance of the animals in the test.

Finally, we tested a cohort of 6-month-old wt and c-rel^-/-^ mice with the odor preference test, to examine whether mice preferred one of the two odors used in the discrimination task. This was not the case, since, when exposed to the more intense vanilla and orange scent (dilution 1:10^4^), wt and c-rel^-/-^ mice spent similar time sniffing the odors (Additional file 1: Figure S1j).

Taken together, these results demonstrated that c-rel^-/-^ mice were impaired in their ability to detect and discriminate odors when compared to wt mice. The olfactory deficits were already detectable in young c-rel^-/-^ mice and progressively increased with age.

### Progressive and diffused α-synuclein accumulation in the brain of c-rel^-/-^ mice

**Fig. 3:**
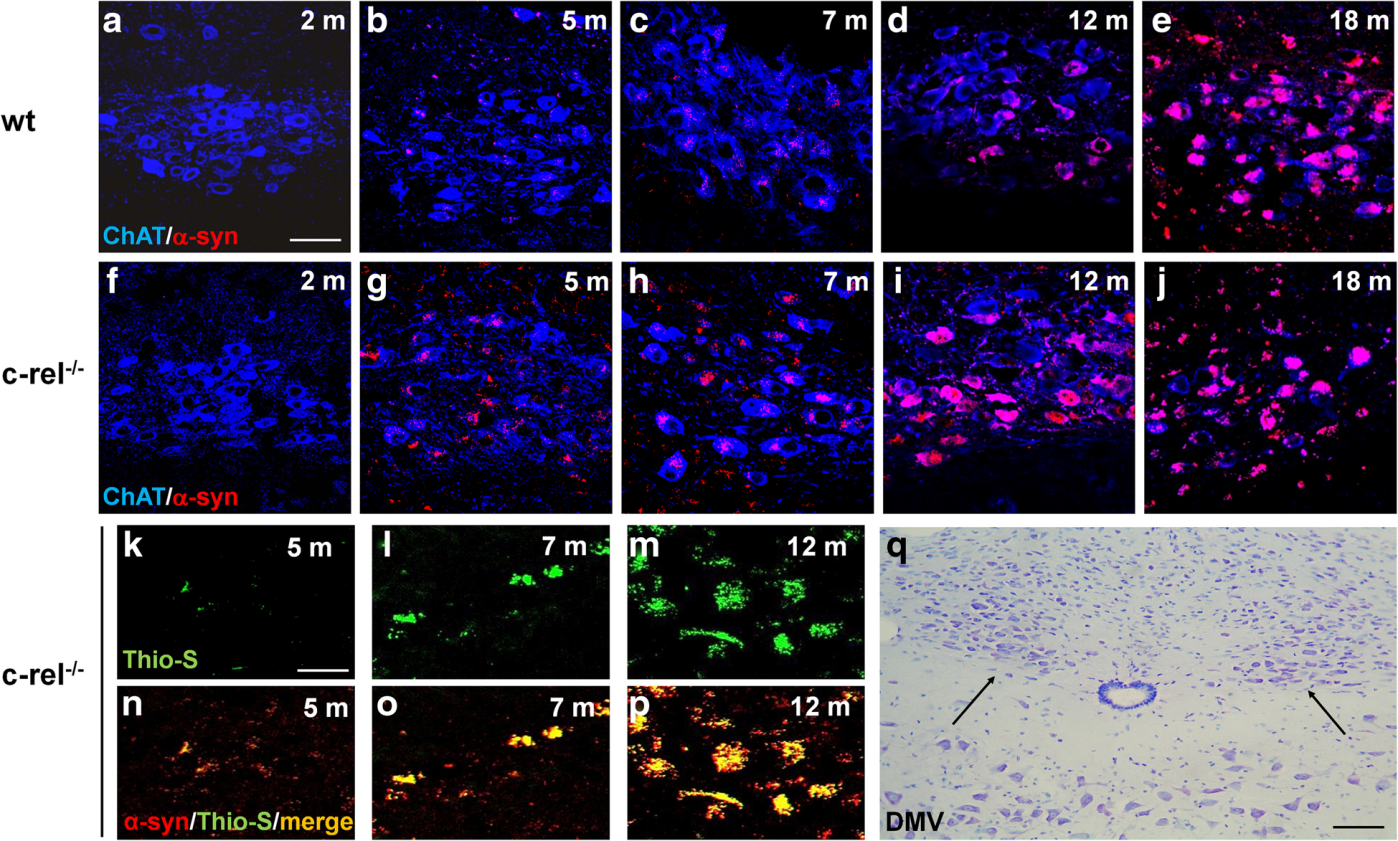
Progressive α-synuclein accumulation in the DMV of c-rel^-/-^ mice. **a**-**j** Representative photomicrographs showing α-synuclein/ChAT double immunofluorescence labeling in 2-, 5-, 7-, 12- and 18-month-old wt and c-rel^-/-^ mice. Please note the earlier appearance and age-related progressive increase of α-synuclein accumulation in the c-rel^-/-^ mice starting from 5 months. *n* = 3 animals per group. **k-p** Representative photomicrographs showing thioflavin-S/α-synuclein double labeling in 5-, 7- and 12-month-old c-rel^-/-^ mice. The yellow signal in the merge is indicative of the presence of fibrillary aggregated α-synuclein in the DMV. *n* = 3 animals per group. **q** Nissl-stained sections showing the DMV area. Scale bars: in **a** = 60 μm for (**a**-**j**); in (**k**) = 30 μm for (**k**-**p**). **q** = 120 μm

We investigated neuronal α-synuclein deposition by fluorescence double labelling in the DMV, LC and SN pars compacta of 2-, 5-, 7-, 12- and 18-month-old wt and c-rel^-/-^ mice. In c-rel^-/-^ mice, α-synuclein accumulation within ChAT-positive neurons of the DMV was nearly detectable at 5 months and progressively increased in older mice (Fig. 3g-j). The early α-synuclein accumulation was confirmed in 7-month-old c-rel^-/-^ mice by quantification of the total immuneactivity (Additional file 3: Figure S3a). The α-synuclein deposits were thioflavin S-positive, supporting the presence of fibrillary aggregates (Fig. 3k-p). In wt mice, the accumulation of fibrillary α-synuclein in the DMV cholinergic neurons was undetectable till 12 months of age (Fig. 3d, e and Additional file 2: Figure S2a, b).

**Fig. 4:**
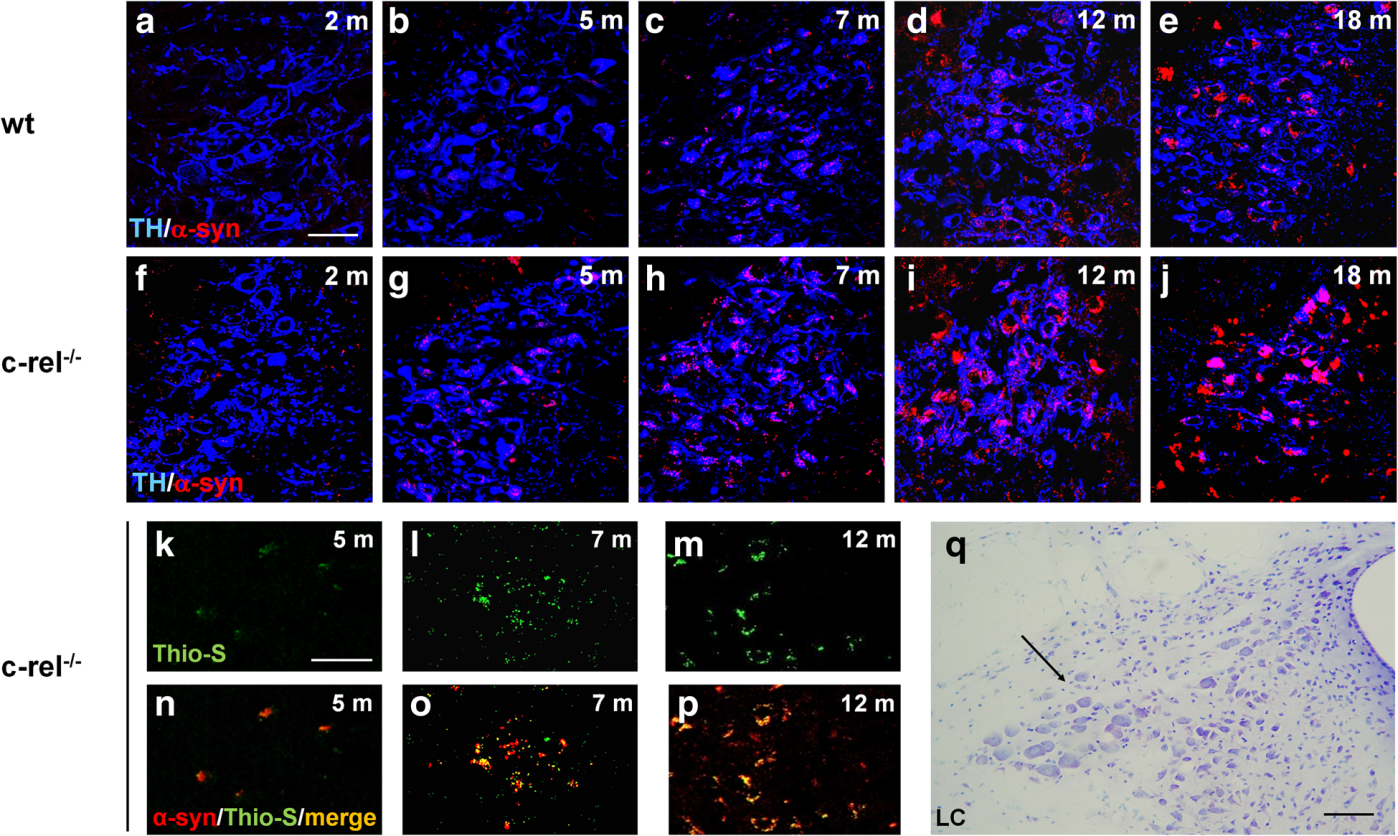
Progressive α-synuclein accumulation in the LC of c-rel^-/-^ mice. **a**-**j** Representative photomicrographs showing α-synuclein/TH double immunofluorescence labeling in 2-, 5-, 7-, 12- and 18-month-old wt and c-rel^-/-^ mice. Alpha-synuclein accumulation in c-rel^-/-^ mice is visible starting from 5 months and increase thereafter. *n* = 3 animals per group. **k-p** Representative photomicrographs displaying thioflavin-S/α-synuclein double labeling in 5-, 7- and 12-month-old c-rel^-/-^ mice. The presence of fibrillary aggregated α-synuclein is revealed by the yellow signal in the merge. *n* = 3 animals per group. **q** Nissl-stained sections showing the LC area. Scale bars: in **a** = 50 μm for (**a-j**); in (**k**) = 80 μm for (**k-p)**. **q** = 120 μm

In the LC of c-rel^-/-^ mice, progressive deposition of fibrillary α-synuclein within TH-positive neurons was detected from 5 months (Fig. 4f-p). The α-synuclein accumulation in c-rel^-/-^ mice was confirmed by quantification of total immunoreactivity at 7 months (Additional file 3: Figure S3b). Conversely, a scattered α-synuclein staining was detected in some of the TH-positive neurons of wt LC only at 18 months of age (Fig. 4a-e).

**Fig. 5:**
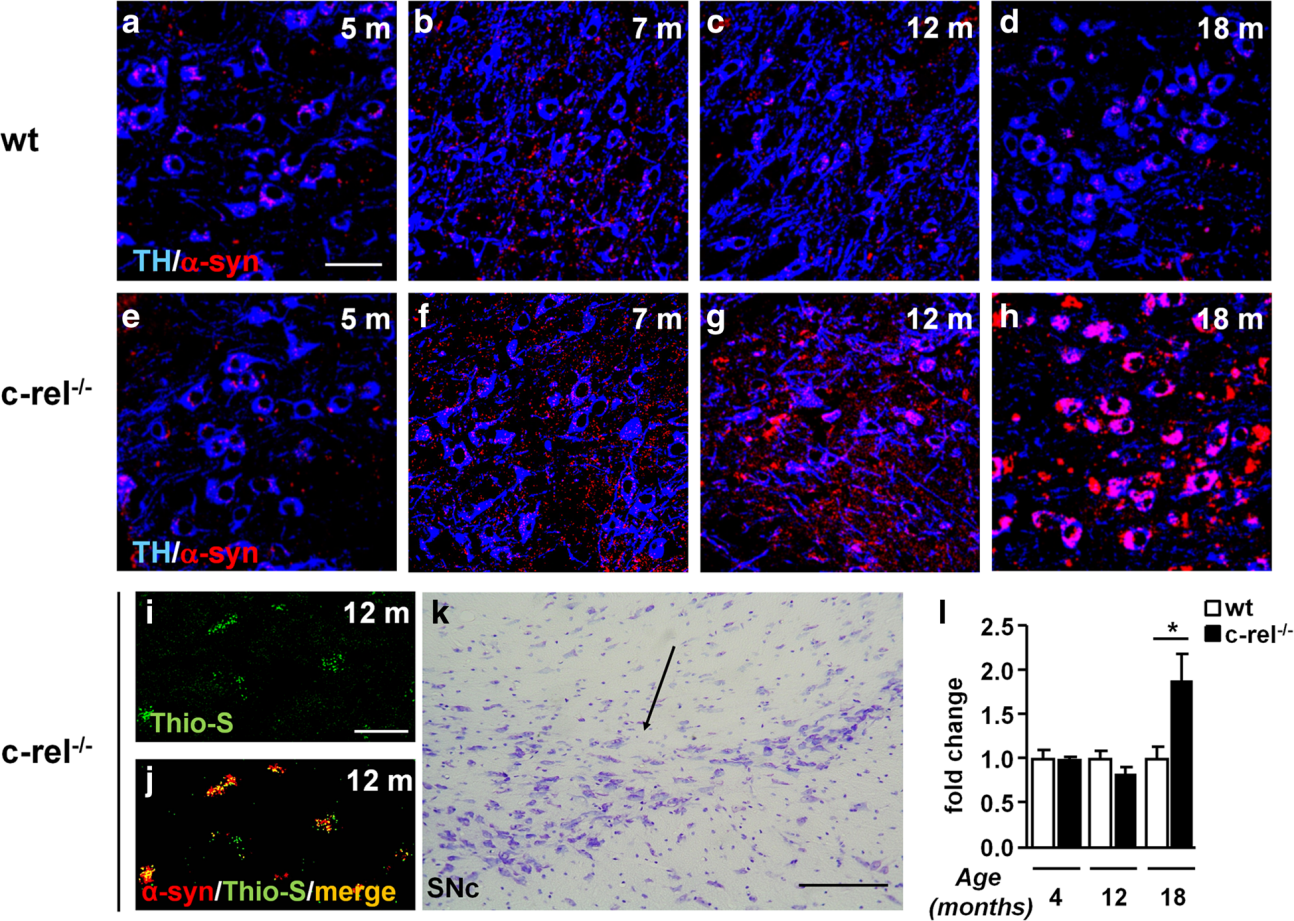
Progressive α-synuclein accumulation in the SN of c-rel^-/-^ mice. **a**-**h** Representative photomicrographs showing α-synuclein/TH double immunofluorescence labeling in the SN pars compacta of 5-, 7-, 12- and 18-month-old wt and c-rel^-/-^ mice. Please note the stronger and age-related increase of α-synuclein signal in 12- and 18-month-old c-rel^-/-^ mice compared to wt mice. *n* = 3 animals per group. **i, j** Representative photomicrographs showing thioflavin-S/α-synuclein double labeling in 12-month-old c-rel^-/-^ mice. The yellow signal in the merge indicates the presence of fibrillary aggregated α-synuclein. *n* = 3 animals per group. **k** Nissl-stained sections showing the SN pars compacta. Scale bars: in **a** = 50 μm for (**a**-**h**); in **i** = 35 μm for (**i**, **j**). **q** = 160 μm. **l** Evaluation of α-synuclein mRNA transcripts in the SN of 4-, 12- and 18-month-old wt and c-rel^-/-^ mice. Alpha-synuclein expression was increased in c-rel^-/-^ mice at 18 months. *n* = 3-6 animals per group, *p<0.05 *vs*. wt mice, t-test

Alpha-synuclein became evident in the SN pars compacta of c-rel^-/-^ mice at 12 months (Fig. 5g) as confirmed by the image analysis (Additional file 3: Figure S3c), and further increased in TH-positive neurons at 18 months (Fig. 5h). The thioflavin-S/α-synuclein double labelling again supported a fibrillary form of accumulated α-synuclein (Fig. 5i-j). In wt mice, the SN pars compacta was spared from α-synuclein deposits till 18 months (Fig. 5a-d), when, in line with previous data [12], only a mild α-synuclein immunoreactivity in a small fraction of TH-positive cells was observed (Fig. 5d).

Further analysis showed that the thioflavin S/α-synuclein-positive inclusions, detected in DMV and LC at 7 months and in the SN pars compacta at 12 months, were proteinase K-resistant, confirming the fibrillary aggregation status of α-synuclein (Additional file 4: Figure S4a-f).It has been found that most of the α-synuclein accumulated in brains of patients is phosphorylated at serine 129 [27, 28]. c-Rel deficient mice also exhibited a mild Pser129-α-synuclein immunoreactivity in DMV and LC at 7 months and in the SN pars compacta, at 12 months (Additional file 4: Figure S4g-i).To investigate whether α-synuclein accumulation in SN was related to increased α-synuclein gene expression, we performed quantitative RT-PCR analysis of transcripts in 4-, 12- and 18-month-old wt and c-rel^-/-^ mice (Fig. 5l). Only 18-month-old c-rel^-/-^ mice showed a significant augment of α-synuclein expression, implicating that the protein accumulation in 12-month-old mice was independent of “*de novo*” protein synthesis.

Dopaminergic neurons in SN pars compacta are “high energy-demanding” cells. They require elevated energy production by mitochondria and concomitantly generate a large amount of reactive oxygen/nitrogen species (ROS/RNS) that need to be constantly neutralized [29, 30]. To correlate α-synuclein pathology to potential mitochondrial dysfunctions, we assessed whether c-Rel deficiency might affect mitochondria homeostasis in SN neurons. To this purpose, we performed quantitative RT-PCR analysis of transcripts for factors contributing to mitochondrial homeostasis (UCP4, UCP5, PGC1α and Bcl-xL [31, 32]) and antioxidant scavenging (MnSOD) in 4-, 12- and 18-month-old mice (Additional file 5: Figure S5a-e). We did not detect differences in the expression of the above genes in c-rel^-/-^ mice at 4 months of age. At 12 months, c-rel^-/-^ mice exhibited a significant decrease of UCP5 (Additional file 5: Figure S5b), paralleled by a marked elevation of PGC1α expression (Additional file 5: Figure S5d). At 18 months, beside UCP5 also UCP4, MnSOD and Bcl-xL were markedly decreased in c-rel^-/-^ mice, while the expression of PGC1α dropped to a level comparable to that of wt littermates.

**Fig. 6:**
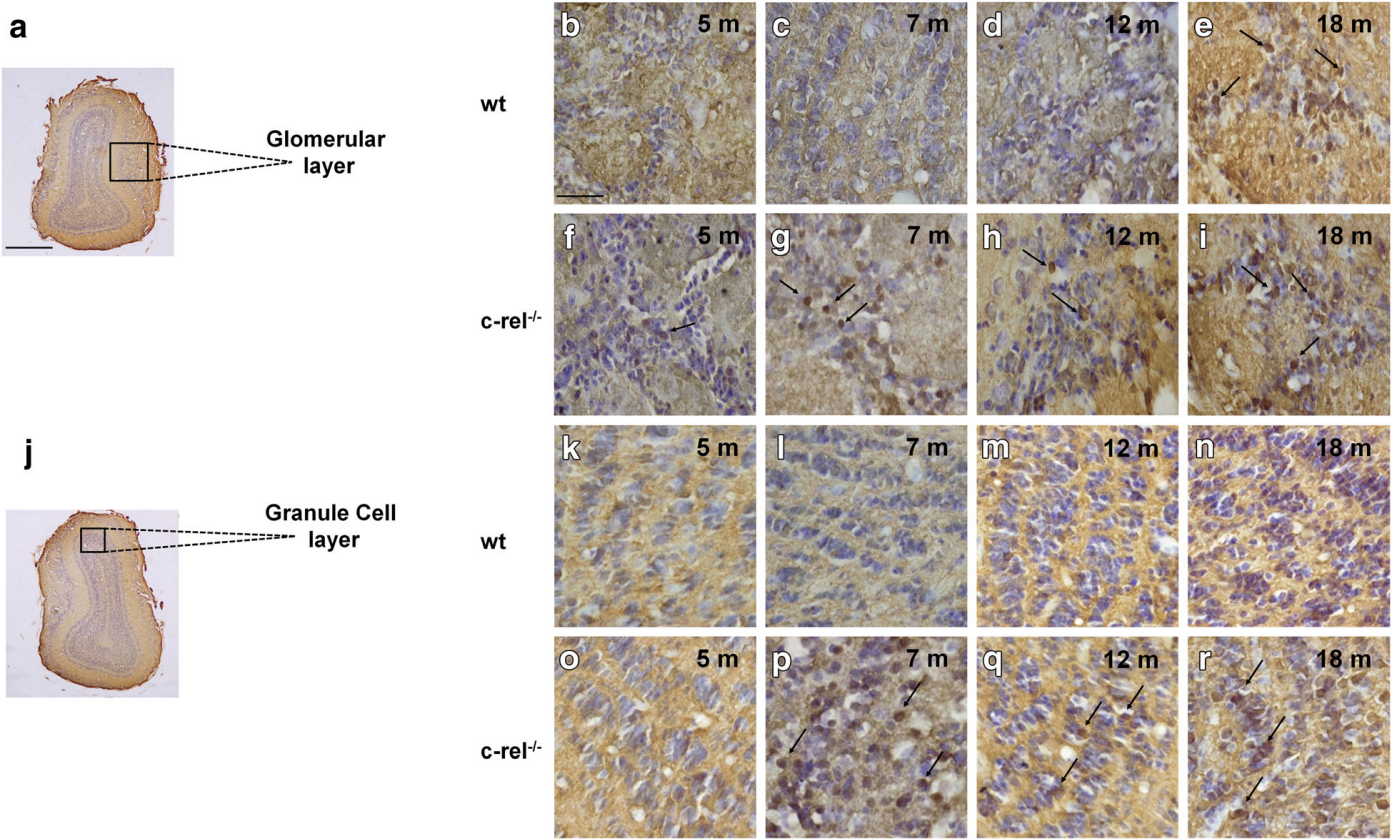
Progressive α-synuclein accumulation in the OB of c-rel^-/-^ mice. **a**, **j** Nissl-stained coronal sections of OB showing glomerular and granule cell layers, respectively. **b-i** and (**k-r**) Representative photomicrographs showing α-synuclein immunoreactivity in the glomerular layer (**b-i**) and granule cell layer (**k-r**) from the OB of 5-, 7-, 12- and 18-month-old wt and c-rel^-/-^ mice. *n* = 3 animals per group. The arrows indicate α-synuclein signal. c-rel^-/-^ mice exhibit earlier and stronger expression of α-synuclein in the different layers of the OB when compared to wt mice. Scale bars: in **a** = 1000 μm for (**a**, **j**); in **b** = 250 μm for (**b**-**i**) and (**k**-**r**)

Finally, we investigated the presence of α-synuclein deposition in the OB of 5-, 7-, 12- and 18-month-old wt and c-rel^-/-^ mice (Fig. 6). A marked α-synuclein immunoreactivity was obtained in glomerular and granule cell layers of the OB of c-rel^-/-^ mice. The protein accumulation progressively increased in both cell layers starting from 5 and 7 months, respectively (Fig. 6f-i and p-r). Wild-type mice only showed minor immunoreactivity restricted to the glomerular layer at 18 months (Fig. 6e). The presence of α-synuclein inclusions in 7-month-old c-rel^-/-^ mice was supported by analysis of the immunoreactive area (Additional file 3: Figure S3d).

### Accumulation of α-synuclein in the distal colon of 2-month-old c-rel^-/-^ mice

**Fig. 7:**
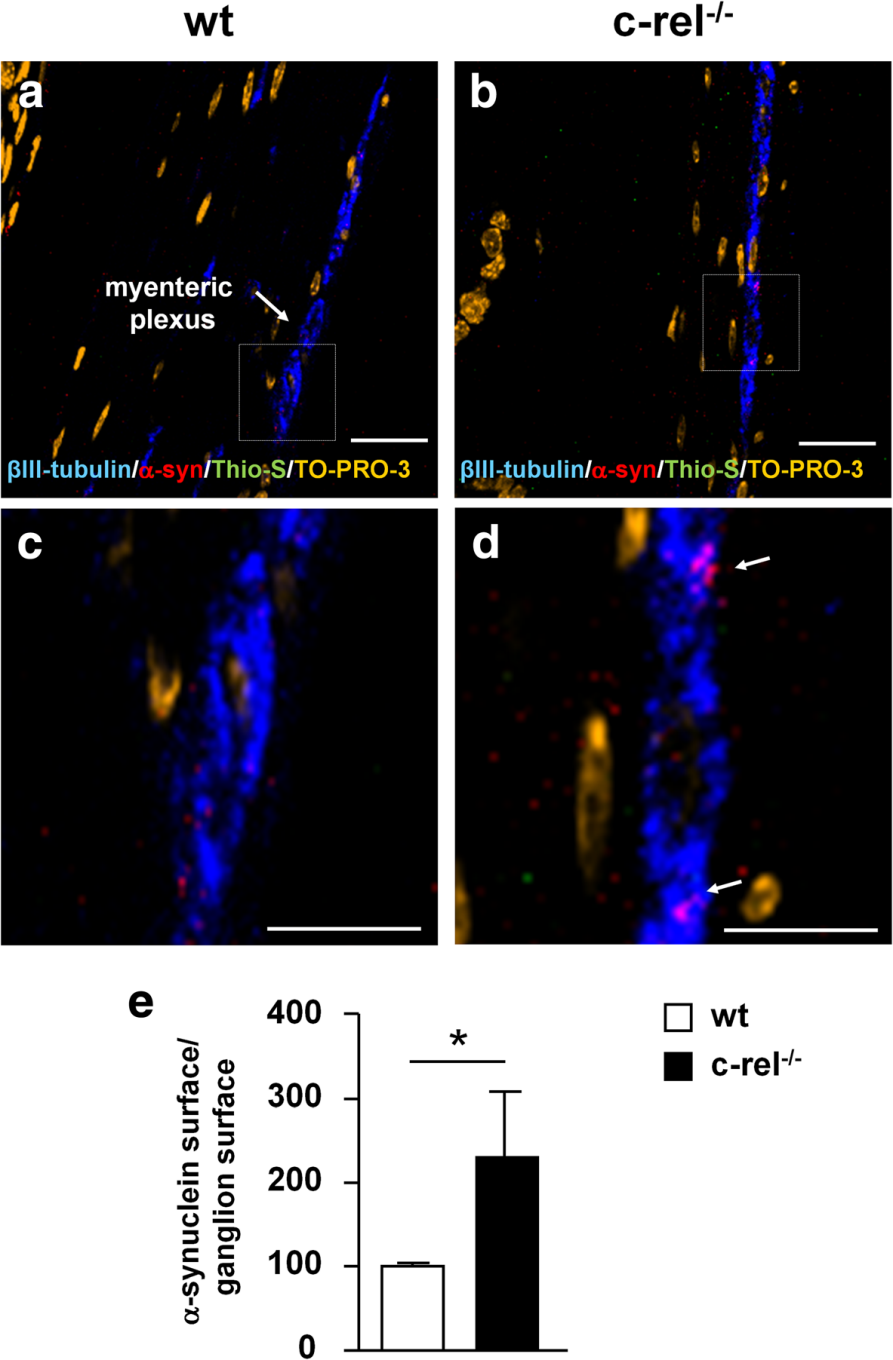
Accumulation of α-synuclein in the distal colon of 2-month-old c-rel^-/-^ mice. Representative photomicrographs displaying thioflavin-S/α-synuclein/βIII-tubulin/TO-PRO-3 immunofluorescence labeling in sections of distal colon from 2-month-old wt and c-rel^-/-^ mice (**a**, **b**). High magnification of the areas in the squares in panel a and b are also provided (**c**, **d**). Please note the presence of bigger α-synuclein-positive inclusions in βIII-tubulin-positive neurons of c-rel^-/-^ mice (arrow in panel **d**) when compared to those observed in wt littermates. This notwithstanding, the α-synuclein-positive inclusions of c-rel^-/-^ were thioflavin-S/negative. **e** Quantification of total α-synuclein-positive surface normalized by the ganglion area confirmed a significant increase of α-synuclein immunoreactivity in c-rel^-/-^ mice when compared to wt littermates. *n* = 5-6 animals per group, *p<0.01, Kolmogorov-Smirnov test. Scale bars: in **a, b** = 20 μm; in **c, d** = 10 μm

We also investigated whether constipation in young c-rel^-/-^ mice at 2 months of age was paralleled by α-synuclein accumulation in the enteric nervous system (ENS) ganglia. To this purpose, we performed a double immunolabeling for α-synuclein and β3-tubulin coupled with Thioflavin-S and TO-PRO-3 staining to assay whether α-synuclein may be accumulated in a fibrillary form in myenteric plexus of the distal colon, which is the gastrointestinal tract most involved in the intestinal motility [33]. We observed a mild accumulation of α-synuclein in colonic myenteric ganglia of 2-month-old c-rel^-/-^ mice (Fig. 7a-d) that was confirmed by a statistically significant increase of total α-synuclein immunoreactivity normalized for ganglion surface (Fig. 7e) *vs* wt littermates. The α-synuclein deposits of c-rel^-/-^ mice resulted thioflavin S/negative, indicating the absence of α-synuclein fibrillation.

### Age-dependent striatal alterations in c-rel^-/-^ mice

**Fig. 8:**
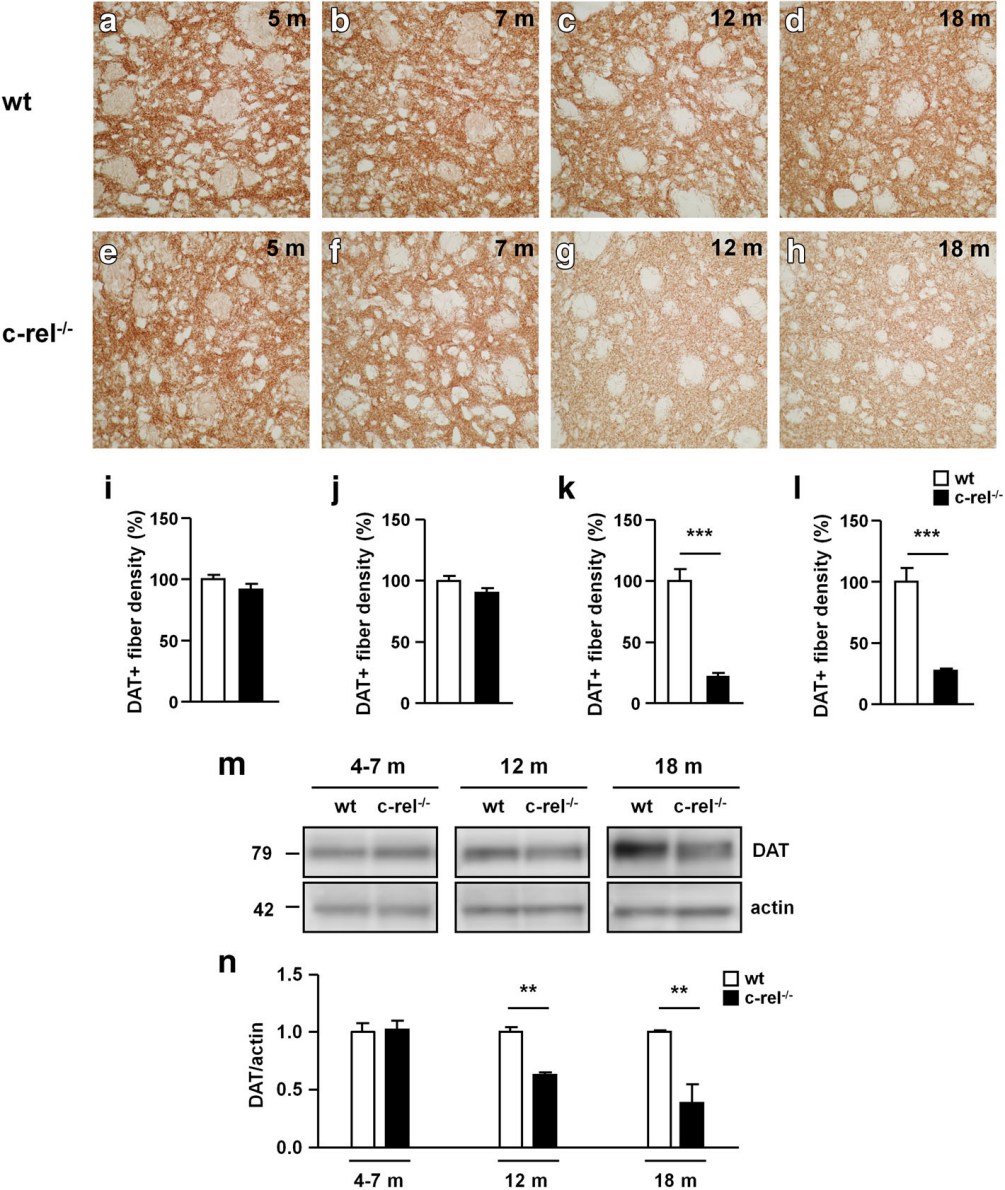
DAT loss in the striatum of c-rel^-/-^ mice occurs before the onset of motor deficits. Representative photomicrographs of DAT positive fibers density in the striatum of wt (**a-d**) and c-rel^-/-^ mice (**e-h**) at 5, 7, 12 and 18 months of age. Densitometric analysis of DAT positive fibers is shown in (**i-l**). The results are expressed as percentage of DAT positive fibers, considering 100% the values obtained for wt mice. Densitometric analysis revealed a significant decrease in the density of DAT positive fibers already in 12-month-old c-rel^-/-^ mice. *n* = 3 animals per group, ***p<0.001 *vs*. wt mice, t-test. **m** Representative immunoblotting of DAT in the striatum of wt and c-rel^-/-^ mice at 4-7, 12 and 18 months. **n** Densitometric analysis confirmed a significant reduction of DAT levels in c-rel^-/-^ mice starting at 12 months. *n* = 6 animals per group, **p<0.01 *vs*. wt mice, t-test

**Fig. 9:**
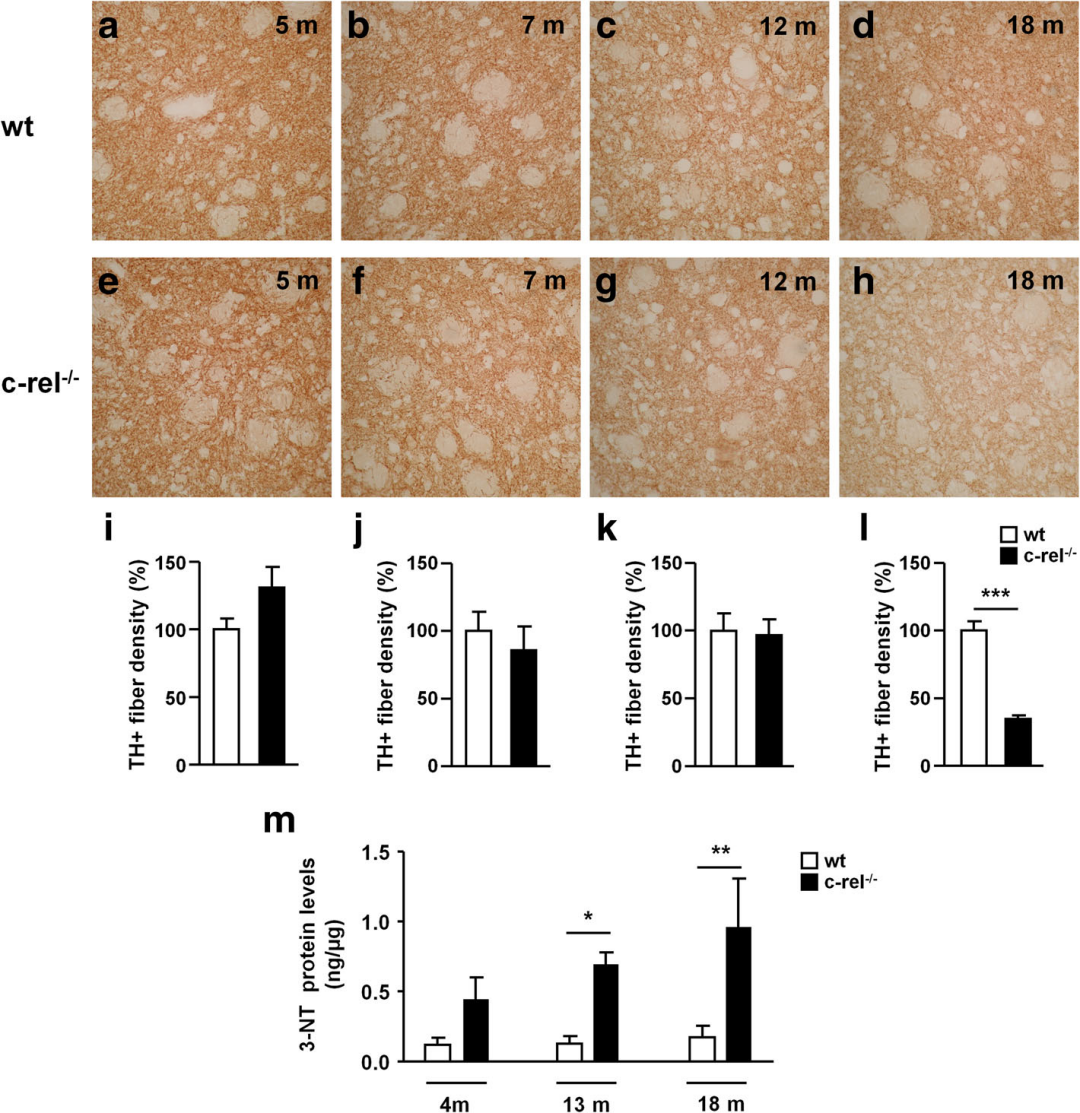
Late loss of nigrostriatal projections in the striatum of c-rel^-/-^ mice. Representative photomicrographs of TH positive fibers density in the striatum of wt (**a-d**) and c-rel^-/-^ mice (**e-h**) at 5, 7, 12 and 18 months of age. Densitometric analysis of TH positive fibers is shown in **(i-l)**. The results are expressed as percentage of TH positive fibers, considering 100% the values obtained for wt mice. A significant reduction in the density of TH positive fibers of c-rel^-/-^ mice was detected only at 18 months*. n* = 3-6 animals per group, ***p<0.001 *vs*. wt mice, t-test. **m** Striatum protein extracts derived from wt and c-rel^-/-^ mice of 4, 12 and 18 months of age have been processed to determine 3-NT protein content. Results are expressed as ng of 3-NT protein amount over μg of total protein extract. 3-NT levels were increased in the striatum of 12-month-old c-rel^-/-^ mice and further increased at 18 months. *n* = 6 animals per group, *p<0.05, *** *p*<0.001 *vs*. wt mice, two way ANOVA followed by Bonferroni *post hoc* test

Among the different pre-motor features of PD, loss of DAT in the striatum is particularly interesting due to its role as a diagnostic marker of parkinsonian degeneration [34]. We evaluated DAT levels in 5-, 7-, 12- and 18-month-old wt and c-rel^-/-^ mice (Fig. 8a-l). Our results showed a marked reduction of DAT immunoreactivity in the striatum of c-rel^-/-^ compared to wt mice from 12 months of age (Fig. 8g, h, k, l). Western blot analysis confirmed decreased levels of striatal DAT starting from 12 months in c-rel^-/-^ mice (Fig. 8m and n). To determine whether the drop of striatal DAT in 12-month-old c-rel^-/-^ mice, showing neither nigral dopamine neuron decrease nor motor impairment yet [5], was associated with a concomitant loss of nigrostriatal projections, we quantified TH-positive nerve fibers in the dorsal striatum (Fig. 9a-l). No differences were detected in the density of striatal TH-positive fibers between wt and c-rel^-/-^ mice at 12 months. Only at 18 months a marked reduction of the area occupied by TH-positive fibers was observed in c-rel^-/-^ mice (Fig. 9h, l), in line with our previous findings [13].

The fact that the reduction of DAT levels is not accompanied by the decrease of TH-positive fibers at 12 months is supportive of the occurrence of a loss of striatal dopaminergic terminals. This was corroborated by double TH/VMAT2 immunofluorescence-based confocal analysis. Indeed, we could detect a shrinkage of striatal VMAT2 immunoreactivity on TH-positive fibers in the striatum of c-rel^-/-^ mice when compared to age-matched controls (Additional file 6: Figure S6).

We then investigated the post-transcriptional modifications induced by ROS/RNS, measuring 3-NT-modified proteins in the striatum of 4-, 12- and 18-month-old wt and c-rel^-/-^ mice (Fig. 9m). In line with the RT-PCR results obtained in the SN (Additional file 5: Figure S5), we observed a progressive increase of 3-NT modified protein levels in the striatum of c-rel^-/-^ mice starting from 12 months, suggesting that the striatal oxidative/nitrosative stress paralleled α-synuclein accumulation in dopamine neuronal soma and the loss of DAT in nerve terminals.

## Discussion

Our results show that constitutive deficiency of NF-κB/c-Rel factor, besides promoting a late-onset parkinsonism [13], generates a prodromal syndrome and a Braak-like stereotyped diffusion of synucleinopathy mimicking sporadic PD.

Constipation is the most common gastrointestinal symptom in PD, reported in more than 80% of affected patients [35] and anticipates motor deficits by 20 years [36]. Functional analysis of the intestine showed that slow colonic transit is the primary cause of constipation in PD [37, 38]. We demonstrated that at 2 months of age, c-rel^-/-^ mice already displayed reduced stool frequency and stool water content. This supports a prolonged time of colonic transit that can be indicative of an early decrease of colon motility. As 2-month-old c-rel^-/-^ mice do not display motor dysfunctions yet [13, 39], we could exclude that the observed early constipation was caused by motor impairment. In addition, c-rel^-/-^ mice did not show differences in food and water intake when compared to wt mice, thus avoiding the possibility that feeding changes might be responsible for the reduced colon motility. A reduced anxiety-like behavior has been associated with a decreased fecal output in mice [40]. However, the absence of a reduced anxiety-like behavior in c-rel^-/-^ mice ruled out the involvement of emotional factors in triggering constipation.

Hyposmia also typically affects idiopathic PD patients [41, 42] and, by preceding the onset of motor deficits [43], it is also considered a prodromal symptom of PD [41, 44].

We found that c-rel^-/-^ mice performed worse than the corresponding age-matched wt in the odor detection test. By using a medium concentration of vanilla odor (dilution 1:10^6^), we detected a lower capability of 2- and 5-month-old c-rel^-/-^ mice in locating the scent when compared to wt mice. The higher odor concentration (dilution 1:10^4^) allowed us to differentiate between the two groups at 12 and 20 months. At these ages, both groups of mice displayed similar values of total sniffing time, suggesting that abnormalities in the exploratory behavior were not involved in the poor performance of c-Rel deficient mice. Also, the lower score of 2- and 12-month-old c-rel^-/-^ mice was not secondary to motor dysfunctions as hypomotility appeared only at 18 months [13, 39]. These data indicate that the progressive, age-dependent hyposmia appeared early in c-rel^-/-^ mice when compared to wt. In the odor detection task, wt mice maintained the capability of identifying medium concentrations of vanilla till 9 months, and more concentrated odors (dilution 1:10^4^) till 20 months. These findings are in line with evidence showing the age-dependent decline of olfaction is a common process in both humans and mice [45].

In addition, c-rel^-/-^ mice were unable to discriminate between familiar vanilla odor and the novel orange scent in the odor and item discrimination test. In accordance with the findings obtained with the odor detection test, young c-rel^-/-^ mice (3-6 months) did not recognize the novel scent at the concentration 1:10^6^. Likewise, aged c-rel^-/-^ animals (16-18 months) did not discriminate the novel scent at a higher odor concentration (dilution 1:10^4^). At early age, c-Rel KO mouse line has been shown to have defects of memory consolidation, as detected after a 24 h trial interval in the novel object recognition task [39]. Though, as shown by their capability to locate the novel object in the odor and item discrimination test after trial intervals of 1 minute, no cognitive deficits in c-rel^-/-^ mice could have interfered with the interpretation of the odor task in that short time.

These findings support that mice lacking c-Rel exhibit age-dependent olfactory impairments occurring as deficits of both odor detection and odor discrimination. The olfactory deficits observed in c-rel^-/-^ mice are consistent with clinical observations reporting reduced ability to detect and discriminate odors in patients affected by PD [44, 46].

When we looked at α-synuclein accumulation in the c-rel^-/-^ brain, we found that it became detectable in OB, DMV and LC from 5 months onwards, while in the SN it appeared from 12 months onwards. In the SN of 12-month-old mice, where a mild proinflammatory transcription has been reported [15], no change in α-synuclein mRNA was found, suggesting that α-synuclein expression does not contribute to the protein accumulation in dopaminergic cells at that stage. The α-synuclein-immunopositive deposits were also proteinase K-resistant and thioflavin S-reactive, indicating that the protein was aggregated in a fibrillary form. Moreover, we also detected the presence of PSer129 α-synuclein, which has been found to promote α-synuclein fibrillation and internalization and can contribute to PD pathogenesis [47–50]. While damages in the DMV and SN could contribute to functional gastrointestinal disturbances [51, 52], biopsy studies showing α-synuclein accumulation in the bowel of pre-clinical PD patients suggest that accumulation of the protein in the ENS underlies the early onset of gut dysfunction [53]. The early α-synuclein accumulation observed in the colonic myenteric ganglia of 2-month-old c-rel^-/-^ mice supports this hypothesis. Studies are currently ongoing by our group to fully characterize synuleinopathy in the ENS of the c-rel^-/-^ mouse model.

As much as hyposmia in PD, α-synuclein accumulation in the olfactory epithelium is proposed to be related to early deficits in odor detection [54]. The discovery of a direct dopaminergic innervation between SN and OB in rats might provide an additional neuroanatomical pathway for the spread of α-synuclein from the OB to basal ganglia [55]. Recent studies have shown that α-synuclein injected in the OB is taken up by neurons, spreads along axons to different brain regions including the LC and SN, and its uptake is coupled with progressive deficits in olfactory function [56, 57]. Further investigation will clarify whether also in c-rel^-/-^ mice α-synuclein accumulation originates from olfactory epithelium.

Together with α-synuclein burden in the SN, we observed a significant reduction of DAT levels in the striatum at 12 months. The drop of DAT occurred in spite of conserved TH-positive neurons [13] and fibers at that age, indicating that changes in nigrostriatal terminals anticipated the nigral neuron degeneration. This finding is in agreement with the clinical prognostic value ascribed to positive DaTSCAN in PD patients. It is also in line with studies showing that α-synuclein overexpression in mice triggers striatal synaptic failure together with a retrograde axonal-to-cell body striatonigral degeneration [58–62]. Since α-synuclein can directly affect DAT and VMAT2 trafficking [24, 63–65], it may thus be feasible that the decrease of DAT and VMAT2 observed in the striatum of c-rel^-/-^ mice is a consequence of α-synuclein aggregation at striatal dopaminergic terminals. Studies are currently ongoing by our group to probe this hypothesis.

Although it remains to be determined how exactly the constitutive c-Rel deficiency can induce progressive α-synuclein accumulation and loss of dopaminergic neurons in SN [13], we found that c-rel^-/-^ mice exhibited changes in the expression of proteins controlling mitochondrial homeostasis (PGC1α and Bcl-xL) [32, 66, 67], ROS generation (UCP4 and UCP5) [31, 68] and ROS scavenging (MnSOD) [69, 70]. The mitochondrial energy-transducing capacity is essential for the maintenance of neuronal function and is preserved by uncoupling proteins UCP4 and UCP5 and antioxidant factors, including MnSOD [71]. PGC1α and PGC1α-dependent genes controlling cellular bioenergetics, have been found under-expressed in laser-captured human dopaminergic neurons and SN transcriptomes from post-mortem PD brain [72]. Gene expression profiling of the SN has also revealed a significant reduction of Bcl-xL and MnSOD transcription in PD [73]. Impairment of energy metabolism and mitochondria redox homeostasis is a hallmark of brain aging, which is amplified in the early stages of neurodegenerative diseases. Since c-Rel positively regulates the expression of UCP4, MnSOD and Bcl-xL [14, 68, 74–77], it may be predicted that, by reducing the levels of those proteins, c-Rel deficiency enhances neuronal accumulation of ROS/RNS during aging [78]. Consistent with findings showing high nitration and nitrosylation of proteins, including α-synuclein and parkin, in PD [79–82], we detected a significant increase of 3-NT-modified proteins in striatal extracts from 12 to 18 months in c-rel^-/-^ mice. Reactive nitrogen species have been found to foster both intracellular accumulation of α-synuclein and its aggregation [83]. Yu and colleagues [84] demonstrated that nitration at tyrosine residues 39, 105 and 108 of α-synuclein induces loss of dopaminergic neurons in the SN of rats. Thus, it can be speculated that dysfunction of mitochondrial antioxidant system in c-Rel deficient mice may contribute to enhance oxygen/nitrogen free radicals and α-synuclein aggregation that, in turn, may induce dopaminergic neurons degeneration. Worth of note, at 18 months, high oxidative stress, severe α-synuclein pathology, with iron and DMT-1 accumulation, and nigrostriatal neuron degeneration are associated with striatal increase of RelA(K310) acetylation [14], a transcriptional pathway regulating DMT1 [85] and pro-apoptotic gene expression [86].

The relevance of mitochondrial dysfunction in α-synuclein accumulation is further supported by studies demonstrating that exposure of rodents to mitochondrial toxins causes a pathological accumulation of α-synuclein in central and peripheral neurons [87–89]. Increased α-synuclein expression in the SN may also contribute to the protein accumulation both in mitochondrial toxin-based models [90–93] and in PD subjects [94, 95]. In line with this evidence, 18-month-old c-rel^-/-^ mice showed increased α-synuclein transcription in the SN. This body of evidence suggests that, along aging, the progressive mitochondrial impairment resulting from c-Rel deficiency could be among the mechanisms promoting α-synuclein deposition first and, later, α-synuclein expression.

The temporal and anatomical pattern of α-synuclein accumulation in c-rel^-/-^ mice, involving OB, DMV, LC and SN, agrees with the disease staging proposed by Braak, that correlates the stereotyped diffusion pattern of LB pathology in PD to the development of symptoms severity [96].

To date, two main hypotheses have been proposed to explain the onset timing for motor and non-motor symptoms as well as the pathological progression observed in PD.

According to the “spreading hypothesis”, sporadic PD starts at peripheral level, in the neurons of nasal cavity and in the neurons of ENS in the gut. From these regions, the pathology is hypothesized to spread to the central nervous system (CNS) following a specific pattern, via the olfactory tract and the vagal nerve, respectively [6–8, 97]. The “spreading hypothesis” has been challenged by evidence indicating that cell-autonomous factors may influence both α-synuclein pathology and neuronal cell death [30]. Hence, a “functional threshold theory” for PD has been proposed [98]. This latter hypothesizes that pathogenic mechanisms, that can trigger α-synuclein pathology simultaneously, distress both central and peripheral neurons. The different threshold to stress, and the diverse functional reserve of affected neuronal networks originating at the PNS or CNS, deeply influence the symptoms onset. Prodromal non-motor signs would manifest in relation to the higher proneness of neurons in PNS, OB and LC to accumulate α-synuclein and their relatively lower functional reserve. The later onset of motor symptoms would be associated with the lower sensitivity of midbrain dopamine neurons to accumulate α-synuclein in response to the stress and the larger functional reserve of basal ganglia circuits. The progressive pattern of α-synuclein pathology and the prodromal parkinsonian phenotype of c-rel^-/-^ mice seem to fit with both the spreading hypothesis and the threshold theory.

## Conclusions

Our results indicate that c-rel^-/-^ mice represent a unique mouse model exploitable to study pathogenic mechanisms contributing to the onset of PD, or test the efficacy of therapeutic approaches at PD premotor stages. These data, when coupled to preliminary results showing reduced c-Rel activity in post-mortem PD SN, suggest that c-Rel dysfunction may contribute to PD and could be involved in disease pathogenesis.

## Additional files

Additional file 1: **Figure S1.** Behavioral studies supplementary data. Daily food (**a**) and water intake (**b**) normalized for 30 grams of body weight (bw) of 2-, 5-, 9-, 15- and 20-month-old wt and c-rel^-/-^ mice (*n* =13-28). No significant differences were found between wt and c-rel^-/-^ mice in any of the considered ages (*p*>0.05, t-test). (**c**, **d**, **e**, **f**) Anxiety-like behavior was evaluated in the open field by measuring the time spent in the central zone, considering 100% the values calculated for wt mice. Open field test was performed on 3- (c), 6- (d), 13- (e) and 20- (f) month-old wt and c-rel^-/-^ mice (*n *= 5-7). No significant differences were found between wt and c-rel^-/-^ mice at 3 (c) and 6 months (d) (*p*>0.05, t-test). At 13 (e) and 20 months (f) c-rel^-/-^ mice spent less time in the central zone compared to wt controls (**p<0.01, t-test). (**g**) Odor detection test was performed on 2-, 5-, 9-, 12- and 20-month-old wt and c-rel^-/-^ mice (n = 13-18). Percentage of time sniffing the odor at the concentration 1:10^8^ is shown. Neither wt nor c-rel^-/-^ mice could locate the scent at any of the analyzed ages (*p*>0.05, one-sample t-test vs chance level). Total sniffing time at odor concentrations 1:10^6^ (**h**) and 1:10^4^ (**i**). Total sniffing time values were calculated for wt and c-rel^-/-^ mice at 2 and 5 (h) or 12 and 20 (i) months (*n *= 13-18). No significant differences were found between wt and c-rel^-/-^ mice in any of the considered ages (*p*>0.05, t-test). (**j**) Odor preference test was performed on 6-month-old wt and c-rel^-/-^ mice using vanilla and orange odors diluted 1:10^4^ (*n* = 6). No significant difference in the percentage sniffing the two odors was found neither in wt nor in c-rel^-/-^ mice (*p*>0.05, two way ANOVA followed by Bonferroni *post hoc* test). (TIF 6976 kb)

Additional file 2: **Figure S2.** Thioflavin S/α-synuclein-positive inclusions are detectable in the DMV of wt mice starting at 12 months of age. Thioflavin-S/α-synuclein double labeling in the DMV of 12-month-old of wt (**a**) and c-rel^-/-^ (**b**) mice. *n* = 3 animals per group. Scale bar = 20 μm. (TIF 1904 kb)

Additional file 3: **Figure S3.** Quantification of α-synuclein immunoreactivity. Histograms showing the quantification of total α-synuclein immunoreactivity in the DMV (**a**) LC (**b**) and OB (**d**) at 7 months of age and in the SN at 12 months (**c**). Mice lacking cRel protein showed a statistically significant increase of α-synuclein levels compared to age-matched wt animals. *n* = 3-8 animals per group, *p<0.05, **p<0.01, t-test. (PDF 260 kb)

Additional file 4: **Figure S4.** Alpha-synuclein in c-rel^-/-^ mice is aggregated and phosphorylated. (**a**-**f**) Representative photomicrographs showing proteinase K-resistant α-synuclein in DMV, LC and SN pars compacta of c-rel^-/-^ mice. (**a**, **d**) Thioflavin-S/α-synuclein double immunofluorescence labeling in the DMV of 7-month-old c-rel^-/-^ mice. (**b**, **e**) Thioflavin-S/α-synuclein double labeling in the LC of 7-month-old c-rel^-/-^ mice. (**c**, **f**) Thioflavin-S/α-synuclein double labeling in the SN pars compacta of 12-month-old c-rel^-/-^ mice. The yellow signal in the merge is indicative of the presence of proteinase K-resistant aggregated α-synuclein. *n* = 2 animals per group. Scale bar = 20 μm. (**g-i**) Representative photomicrographs showing Pser129-α-synuclein (P- α-syn) immunoreactivity in DMV, LC and SN pars compacta of c-rel^-/-^ mice. (**g**) Pser129-α-synuclein/ChAT double immunofluorescence labeling in the DMV of 7-month-old c-rel^-/-^ mice. (**h**) Pser129-α-synuclein/TH double immunofluorescence labeling in the LC of 7-month-old c-rel^-/-^ mice. (**i**) Pser129-α-synuclein/TH double immunofluorescence labeling in the SN pars compacta of 12-month-old c-rel^-/-^ mice. Please note that c-Rel deficient mice displayed a mild Pser129-α-synuclein immunoreactivity in the above brain regions. *n* = 3-4 animals per group. Scale bar = 10 μm. (TIF 2826 kb)

Additional file 5: **Figure S5**. Gene expression analysis of proteins governing mitochondrial homeostasis, ROS production and antioxidant scavenging in the SN of wt and c-rel^-/-^ mice. Evaluation of the mRNA transcripts for UCP4 (**a**), UCP5 (**b**), MnSOD (**c**), PGC1α (**d**) and Bcl-xL (**e**) in the SN of 4-, 12- and 18-month-old wt and c-rel^-/-^ mice. No significant differences in the levels of the analyzed transcripts was detectable between wt and c-rel^-/-^ mice at 4 months of age (**a**-**e**). At 12 months c-rel^-/-^ mice exhibited a significant decrease of UCP5 expression (**b**) as well as a significant elevation of PGC1α expression (**d**). At 18 months, beside UCP5, also UCP4, MnSOD and Bcl-xL were significantly diminished in c-rel^-/-^ mice (**a**, **b**, **c**, **e**), while the expression of PGC1α was comparable to that of wt littermates (**d**). *n* = 3-6 animals per group, **p<0.01, ***p<0.001, Student’s t-test. (PDF 2220 kb)

Additional file 6: **Figure S6**. Twelve-month-old c-rel^-/-^ mice display reduced VMAT2 immunoreactivity on striatal TH-positive fibers. Representative photomicrographs showing TH/VMAT-2 double immunofluorescence labeling in the striatum of 12-month-old wt and c-rel^-/-^ mice. The arrows indicate VMAT-2 signal. Please note the lower VMAT-2 signal in c-rel^-/-^ mice. *n* = 5 animals per group. Scale bars: **a**-**f** = 10 μm; **g** and **h** = 2 μm. (TIF 9788 kb)

## Abbreviations

3-NT: 3-nitrotyrosine
Bcl-xL: B-cell lymphoma-extra large
bw: body weight
ChAT: Choline acetyl transferase
CNS: Central nervous system
DAB: 3,3’-diaminobenzidine
DAT: Dopamine transporter
DMT1: Divalent metal transporter 1
DMV: Dorsal motor nucleus of the vagus
ENS: Enteric nervous system
LB: Lewy bodies
LC: Locus coeruleus
OB: Olfactory bulbs
PCG1α: Peroxisome proliferator-activated receptor gamma coactivator 1-α
PD: Parkinson’s disease
PNS: Peripheral nervous system
RBD: Rapid eye movement sleep behaviour disorder
RNS: Reactive nitrogen species; ROS: reactive oxygen species
SEM: Standard error of the mean
SN: Substantia nigra
SOD: Superoxide dismutase
TH: Tyrosine hydroxylase
UCP: Uncoupling protein
VMAT2: Vesicular monoamine transporter 2
wt: wild-type
