# Supplementary figures and images for: NF-κB/c-Rel deficiency causes Parkinson’s disease-like prodromal symptoms and progressive pathology in mice
Source: Transl Neurodegener. 2019 May 21;8:16. doi: 10.1186/s40035-019-0154-z (PMC6530198; doi:10.1186/s40035-019-0154-z)

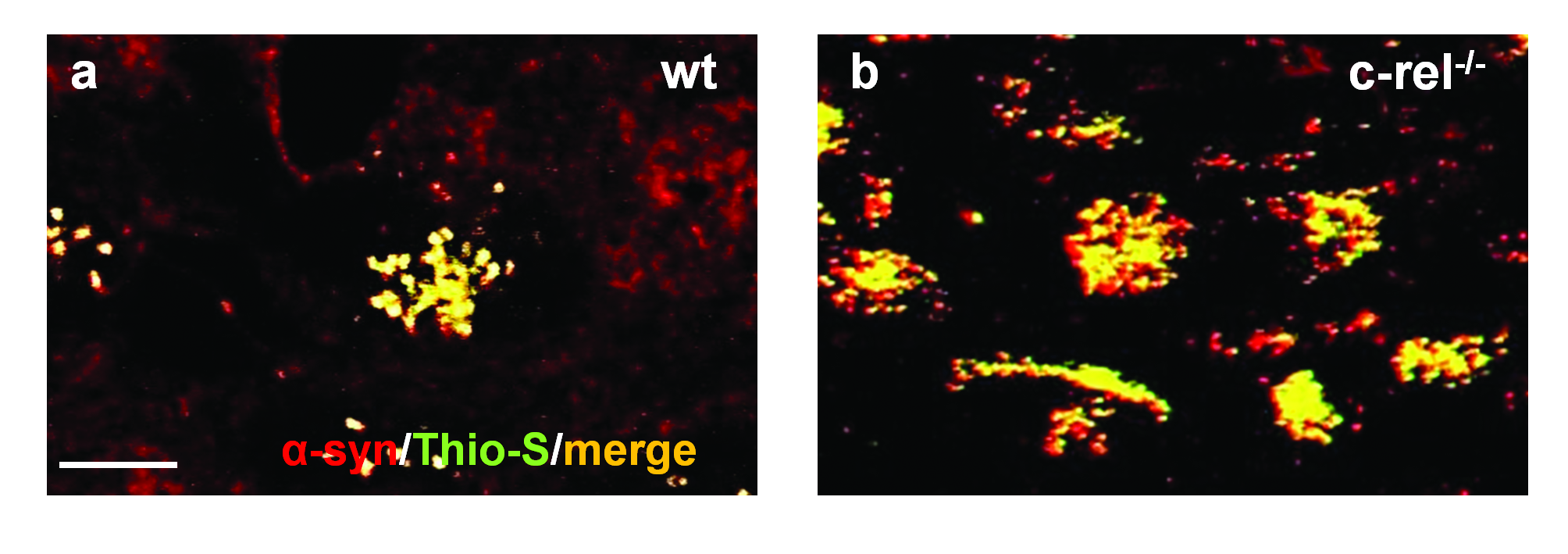

Supplement: Supplementary file 2 — Figure S2. Thioflavin S/α-synuclein-positive inclusions are detectable in the DMV of wt mice starting at 12 months of age. Thioflavin-S/α-synuclein double labeling in the DMV of 12-month-old of wt (a) and c-rel-/- (b) mice. n = 3 animals per group. Scale bar = 20 μm. (TIF 1904 kb) [file 40035_2019_154_MOESM2_ESM.tif]

**a**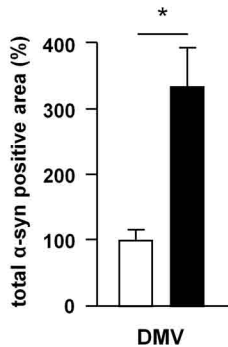**b**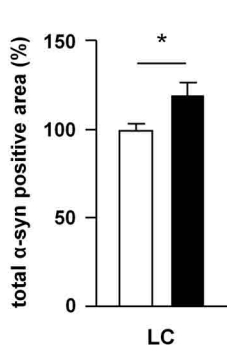**c**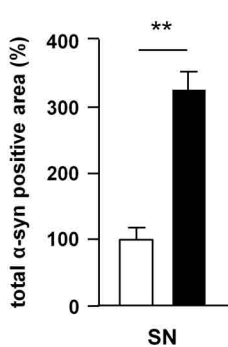**d**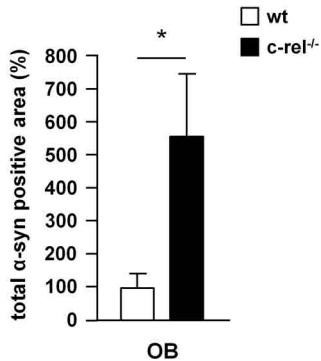

Supplement: Supplementary file 3 — Figure S3. Quantification of α-synuclein immunoreactivity. Histograms showing the quantification of total α-synuclein immunoreactivity in the DMV (a) LC (b) and OB (d) at 7 months of age and in the SN at 12 months (c). Mice lacking cRel protein showed a statistically significant increase of α-synuclein levels compared to age-matched wt animals. n = 3-8 animals per group, *p<0.05, **p<0.01, t-test. (PDF 260 kb) [file 40035_2019_154_MOESM3_ESM.pdf]

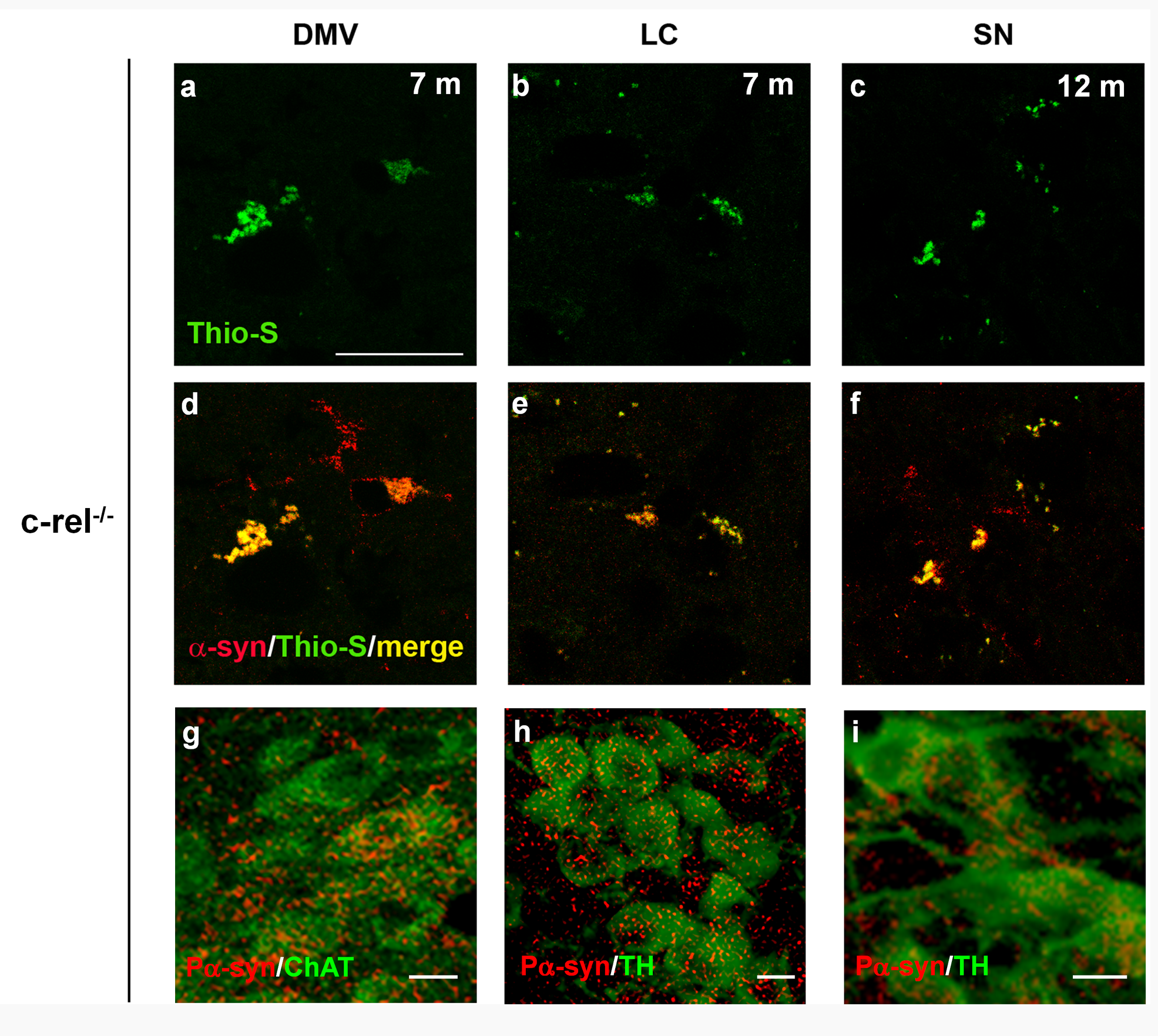

Supplement: Supplementary file 4 — Figure S4. Alpha-synuclein in c-rel-/- mice is aggregated and phosphorylated. (a-f) Representative photomicrographs showing proteinase K-resistant α-synuclein in DMV, LC and SN pars compacta of c-rel-/- mice. (a, d) Thioflavin-S/α-synuclein double immunofluorescence labeling in the DMV of 7-month-old c-rel-/- mice. (b, e) Thioflavin-S/α-synuclein double labeling in the LC of 7-month-old c-rel-/- mice. (c, f) Thioflavin-S/α-synuclein double labeling in the SN pars compacta of 12-month-old c-rel-/- mice. The yellow signal in the merge is indicative of the presence of proteinase K-resistant aggregated α-synuclein. n = 2 animals per group. Scale bar = 20 μm. (g-i) Representative photomicrographs showing Pser129-α-synuclein (P- α-syn) immunoreactivity in DMV, LC and SN pars compacta of c-rel-/- mice. (g) Pser129-α-synuclein/ChAT double immunofluorescence labeling in the DMV of 7-month-old c-rel-/- mice. (h) Pser129-α-synuclein/TH double immunofluorescence labeling in the LC of 7-month-old c-rel-/- mice. (i) Pser129-α-synuclein/TH double immunofluorescence labeling in the SN pars compacta of 12-month-old c-rel-/- mice. Please note that c-Rel deficient mice displayed a mild Pser129-α-synuclein immunoreactivity in the above brain regions. n = 3-4 animals per group. Scale bar = 10 μm. (TIF 2826 kb) [file 40035_2019_154_MOESM4_ESM.tif]

**a****UCP4**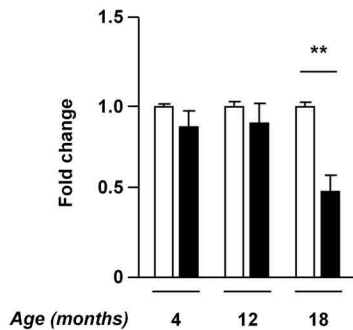**b****UCP5**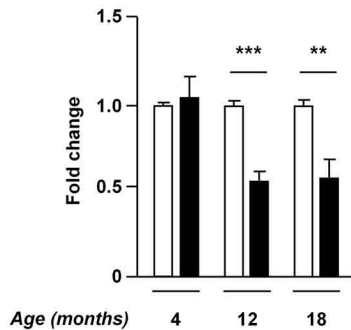**c****MnSOD**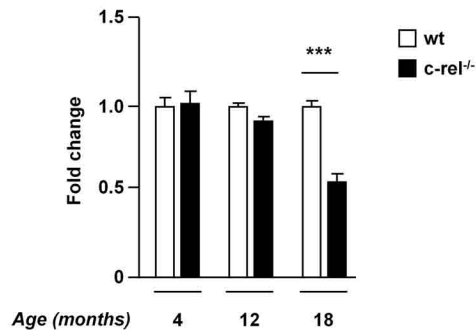**d****PGC1 $\alpha$** 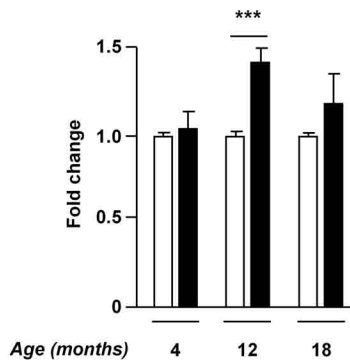**e****Bcl-xL**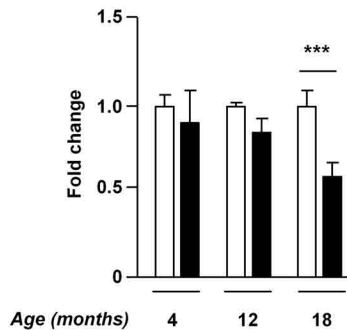

Supplement: Supplementary file 5 — Figure S5. Gene expression analysis of proteins governing mitochondrial homeostasis, ROS production and antioxidant scavenging in the SN of wt and c-rel-/- mice. Evaluation of the mRNA transcripts for UCP4 (a), UCP5 (b), MnSOD (c), PGC1α (d) and Bcl-xL (e) in the SN of 4-, 12- and 18-month-old wt and c-rel-/- mice. No significant differences in the levels of the analyzed transcripts was detectable between wt and c-rel-/- mice at 4 months of age (a-e). At 12 months c-rel-/- mice exhibited a significant decrease of UCP5 expression (b) as well as a significant elevation of PGC1α expression (d). At 18 months, beside UCP5, also UCP4, MnSOD and Bcl-xL were significantly diminished in c-rel-/- mice (a, b, c, e), while the expression of PGC1α was comparable to that of wt littermates (d). n = 3-6 animals per group, **p<0.01, ***p<0.001, Student’s t-test. (PDF 2220 kb) [file 40035_2019_154_MOESM5_ESM.pdf]
